# The Algorithmic Agent Perspective and Computational Neuropsychiatry: From Etiology to Advanced Therapy in Major Depressive Disorder

**DOI:** 10.3390/e26110953

**Published:** 2024-11-06

**Authors:** Giulio Ruffini, Francesca Castaldo, Edmundo Lopez-Sola, Roser Sanchez-Todo, Jakub Vohryzek

**Affiliations:** 1 Brain Modeling Department, Neuroelectrics, 08035 Barcelona, Spain; edmundo.lopez@neuroelectrics.com (E.L.-S.); roser.sanchez@neuroelectrics.com (R.S.-T.); 2 Computational Neuroscience Group, UPF, 08005 Barcelona, Spain; jakub.vohryzek@upf.edu; 3 Centre for Eudaimonia and Human Flourishing, Linacre College, University of Oxford, Oxford OX3 9BX, UK

**Keywords:** algorithmic information theory, computational neuroscience, depression, kolmogorov theory of consciousness, biotypes, brain stimulation, tDCS, artificial intelligence, free energy principle, active inference, digital twins

## Abstract

Major Depressive Disorder (MDD) is a complex, heterogeneous condition affecting millions worldwide. Computational neuropsychiatry offers potential breakthroughs through the mechanistic modeling of this disorder. Using the Kolmogorov theory (KT) of consciousness, we developed a foundational model where algorithmic agents interact with the world to maximize an Objective Function evaluating affective valence. Depression, defined in this context by a state of persistently low valence, may arise from various factors—including inaccurate world models (cognitive biases), a dysfunctional Objective Function (anhedonia, anxiety), deficient planning (executive deficits), or unfavorable environments. Integrating algorithmic, dynamical systems, and neurobiological concepts, we map the agent model to brain circuits and functional networks, framing potential etiological routes and linking with depression biotypes. Finally, we explore how brain stimulation, psychotherapy, and plasticity-enhancing compounds such as psychedelics can synergistically repair neural circuits and optimize therapies using personalized computational models.

## 1. Introduction

Psychiatric disorders account for 23% of the global burden of diseases, with depression as the leading cause [1]. Major Depressive Disorder (MDD) is a pervasive and debilitating mental health condition affecting hundreds of millions of individuals worldwide, characterized by a persistent first-person experience of sadness, hopelessness, and a lack of interest or pleasure in activities (anhedonia) [2,3]. Globally, an estimated 5% of adults suffer from depression, with a higher prevalence among women than men [4]. Furthermore, the incidence of MDD has increased significantly among the young in recent years [5], and adolescents with MDD are up to 30 times more likely to commit suicide [6] (more than 700,000 people die due to suicide every year; it is the fourth leading cause of death in 15–29-year-old individuals [4]). In addition to the considerable burden on personal welfare, depression is accompanied by substantial costs, including health care expenses, as well as less work productivity and higher mortality risk.

MDD is not a single disorder but a highly heterogeneous, multifactorial, and complex phenotype [7]. It encompasses a variety of different disorders, each with its unique causes, symptoms, etiological routes, and treatment responses [8], sometimes referred to as the “spectrum concept” of depression [9]. The symptoms of MDD can vary widely among individuals, with differences in severity, frequency, and duration. This variability is compounded by the frequent co-occurrence of other conditions, such as anxiety disorders or substance use disorders, which can further influence the presentation and course of MDD. As a result, the current definition of depression often leads to a low diagnostic agreement among practitioners, especially when compared to other psychiatric conditions [6].

The limited progress in treating MDD can be attributed to these foundational issues in methodology and theory [7]. Recent research has demonstrated that depression and anxiety patient profiles can be segregated into distinct biotypes. These biotypes, as we discuss below, are characterized by differences in symptoms, behavioral performance on general and emotional cognitive computerized tests, and responses to both pharmacotherapy and behavioral therapy [10,11,12]. This evidence strongly suggests that a personalized approach to treatment, which considers the specific symptom profile and underlying causes for each individual, is essential for effective intervention [12].

While the effects of antidepressants like escitalopram are modest [1] and group-specific, emerging research in the fields of non-invasive brain stimulation, such as transcranial magnetic stimulation (TMS) [13] or transcranial direct current stimulation (tDCS) [14,15], and serotonergic psychedelics [16] provide promising avenues for novel therapeutic strategies. However, the underlying mechanisms governing these interventions and their possible synergistic effects are yet to be fully explored and understood. In particular, while non-invasive brain stimulation can modify brain dynamics and elicit plastic effects, psychedelics may further enhance circuital effects by boosting plasticity and increasing brain dynamics complexity, rendering the system more receptive and malleable to stimulation [17].

To maximize the chance for therapeutic success, it is important to start from a mechanistic framework for both illness and therapy [18]. The complex etiology and network character of MDD motivate the development of whole-brain computational models of this disorder [19,20]. The vision is the synthesis of digital twins or *neurotwins*, computational models of the patient’s brain [21,22,23] representing both physical and network/circuital aspects at multiple scales. A neurotwin can be personalized by assimilating structural and functional neuroimaging data and is designed to reflect the pathology of the patient with sufficient detail for developing personalized, optimized treatments, which can include brain stimulation and pharmacological interventions [24,25,26].

Here, we propose to bridge the historical gap between neurology and psychiatry, underscoring their interconnectedness in understanding mental health disorders. By recognizing the interplay between the brain’s physical structure (akin to computer hardware) and mental processes (software and dynamics), we aim to develop a unified model of MDD integrated with the systems of neuroscience, neuropsychiatry, algorithmic information theory, and dynamical systems theory. This will facilitate the identification of affected circuits to dissect MDD etiology. In this work, we focus on implementing this approach within the *algorithmic agent framework* (or agent framework in short), as defined in the Kolmogorov or algorithmic information theory of consciousness (KT) [27,28]. This perspective emphasizes the importance of integrating computation (mathematics), neuroscience (third-person perspective), and first-person experience when studying depression and other neuropsychiatric conditions, as they offer essential insights for guiding research and clinical applications.

Remarkably, the agent framework offers a transformative approach in neuroscience, akin to viewing the human body through its distinct but interconnected systems, or analyzing an artificial system like an airplane via its electrical, avionics, and power systems. The same analogy can be made with the analysis of well-designed code, which can gain a lot from its modularization. If successful, a subdivision into subsystems/submodules separates functions and minimizes interdependencies while maximizing comprehensibility. As we shall see, the agent framework guides the dissection of complex neural processes into simply defined elements: the Modeling Engine, the Objective Function, and the Planning Engine. Each element corresponds to specific brain regions and circuits, facilitating a more granular understanding of neural functions. Furthermore, the universality of the agent model makes it a powerful tool for conceptual unification, bridging biology and evolution, and extending its applicability to artificial intelligence.

Within this framework, we characterize depression as a disorder of the agent rooted in the malfunction processes influencing the complex process of valence evaluation. We posit that the complexity and diversity inherent to depressive conditions reflect the intricate, multisystem nature of valence evaluation itself. This distinguishes depression from conditions like epilepsy or cancer, where negative valence (reduced well-being) may be evaluated correctly in response to external life events (e.g., disease). We conjecture that the complexity of depression stems from the multiplicity and diversity of systems involved in valence evaluation.

**Figure 1:**
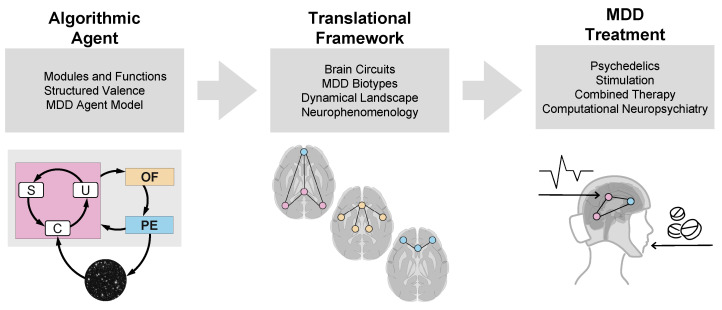
Conceptual Roadmap—From agent theory to treatment. The algorithmic agent framework provides conceptual links between first-person experience and information processing and guides the search for the causes of MDD (see Section 2). The translational framework represents the various theoretical functional modules from the perspective of circuits and dynamics with special emphasis on the clinical biotypes pertaining to MDD. Based on brain circuits and individualized biotypes, different MDD interventions are proposed for treatment.

Connecting third and first-person perspectives, we propose a scientific triangulation [29] among neural, behavioral, and experiential viewpoints that span across both species and the natural-to-artificial divide stemming from the concept of *structured experience* and *valence*—which in KT we associate with the structure of world models and output of the Objective Function. As we discuss in more detail in the next sections, these concepts provide the scaffold to analyze the etiology of depression in humans. This approach, represented in Figure 1, reflects the vision and roadmap for our analysis and connects this abstract perspective with neurobiology, systems neuroscience, and the dynamical perspective. With this basis, we discuss strategies for stratification and model-based personalized treatment of patients using non-invasive brain stimulation combined with neuroplastogens such as psychedelics. As an added benefit of this interdisciplinary exercise, our integrated approach will allow us to extend our understanding of the etiology and phenomenology of MDD to other natural or artificial agents.

Finally, in this paper, we focus on human agents (patients). It is important to clarify that the term *agent* is not used in a derogatory manner toward individuals experiencing MDD. Rather, it refers to a technical concept central to an algorithmic framework for modeling and decision-making.

## 2. The Algorithmic Agent Model

The exploration of consciousness is an age-long topic of profound interest and complexity. The subjective nature of consciousness, often described as the “what it is like to be” experience, alongside its organized nature, presents significant challenges for empirical study. In earlier work, we provided a unifying framework to study consciousness in natural and artificial systems based on algorithmic information theory (AIT)—which we refer to as the Kolmogorov theory (KT) for its foundations on Kolmogorov complexity. Within this framework, we suggest that the brain’s primary role as an information compressor [30,31] gives rise to our structured experience of reality [27,28,32]. We further hypothesized that the successful comparison of models with empirical data generates the qualia of structured experience in computational systems, referred to as *agents* (for an overview of the metaphysical foundations of the theory, see Appendix E).

**Definition 1**  (Algorithmic Agent). *An algorithmic agent is an information-processing system with an Objective Function that interacts bidirectionally with the external world, creating and running compressive models, planning, and acting to maximize its Objective Function.*

Our definition of agent is inspired by natural agents—information-processing systems equipped with elements critical for evolutionary endurance such as *homeostasis* (preservation of self, from the Greek “staying the same across distance” in time) and *telehomeostasis* (preservation of kin, from the Greek “staying the same across distance” in time), or more simply *stasis*. These terms describe the emergent, goal-directed behavior of agents to maintain the stability of their algorithmic pattern over time. Unlike homeostasis, with connotations to reactive stability [33], or allostasis (adaptive prediction) [34], telehomeostasis refers to the intentional drive to preserve an algorithmic pattern through active maintenance. In contrast to autopoiesis, which focuses on self-reproduction [35], telehomeostasis emphasizes long-term persistence through both homeostasis and copying. The definition is central to our analysis of MDD, as it underscores the role of information processing, compression, and planning to subserve the agent’s goals, giving rise to the emergence of structured experience and emotion. Examples of agents include animals, plants, and life in general. The definition is also directly applicable to artificial systems [28]. Indeed, the notion of agent we use here parallels the ones in the foundational literature of artificial intelligence [36]. In Russel and Norvig (1995), an agent is defined as “anything that can be viewed as perceiving its environment through sensors and acting upon that environment through actuators” and a “rational agent” as one that “acts so as to maximize the expected value of a performance measure based on past experience and knowledge” [36]. In their terminology, our algorithmic agent can be seen as a utility-based agent that uses data to build models of the world. In the context of KT, agents are further motivated as an interesting minimalistic solution to the problem of “persistence” of programs in a computational soup [28]). A formal algorithmic specification of the agent is provided in Appendix B, and the relation of the agent framework to the perspective of embodied cognition is discussed in Appendix F.

The agent, as a consequence of creating and running models of the (external) world and itself, is a system with high mutual algorithmic information with its environment [27,28].

**Figure 2:**
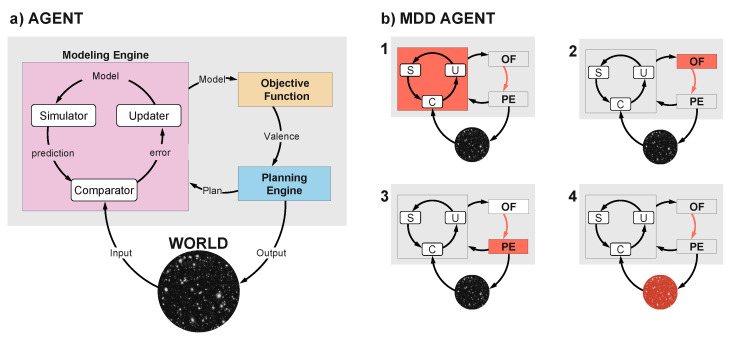
(**a**) Generic agent model. The agent interacts dynamically with its environment and is involved in continuously exchanging data with the external world. The modeling engine runs the model and makes predictions of future data (both from external interfaces and the agent’s own actions). Then, the prediction error (from the Comparator) is evaluated to update the model. The Updater receives prediction errors from the Comparator to improve the model. The Simulator is a shared infrastructure used to run simulations for planning or valence evaluation. The Planning Engine runs counterfactual simulations and selects plans for the next actions (agent outputs). A key agent element is the Objective Function, which the agent aims to optimize through a choice of afferent actions selected by the Planning Engine. (**b**) MDD agent. Non-exclusive dysfunction across the main agent components (**b1**–**b3**) or hostile world inputs (**b4**) can result in sustained low output values from the Objective Function (low valence, red arrow). Identifying those can help us identify the routes that lead the agent to a depressed state.

It can be deconstructed into a set of minimal interconnected modules necessary for stasis [27], each playing a crucial role in the agent’s function: a *Modeling Engine*, an *Objective Function*, and a *Planning Engine* (see Figure 2 and the discussion in Section 2.2). These may be realized in artificial or biological systems in a holistic, distributed, and hierarchical manner involving brain networks and complex multistable dynamics. Their implementation as neural networks, however, is also expected to make them difficult to differentiate. As discussed in [37], the theoretical framework formalizes the notion of such modules. For example, the regulator theorem, a key result in the field of cybernetics, states that “Every good regulator of a system must be a model of that system” [38,39]. In other words, to effectively control a system, the regulator must have a representation or model of the system that captures its essential characteristics. The model, built through the assimilation of past data across timescales, including that from sensors and/or agent actions (see, e.g., [40]), allows the regulator to predict the system’s behavior and adjust its control actions accordingly. In the language of AIT, we express the same by saying that agent and world have a high mutual algorithmic information. The model could be explicitly encoded or inherently integrated within the natural agent’s design—a distinction that dissolves within the realm of algorithmic entities. Similarly, if an agent has to act, choosing an action among many may be seen as optimizing an objective or value function. The agent’s *external interface* or “information membrane” enables its interaction with the external world, mediating both inputs received via sensors and outputs directed to the environment through actuators or effectors. In the Free Energy Principle formulation, this is captured by the notion of a Markov blanket [41,42]—a set of variables that insulates a set of target variables (the agent or world) from the rest of the variables in the system.

In KT, agents enjoy *structured experience*, or S, a central theory concept that bridges the first and third-person views of cognition. This is defined as the spatial, temporal, and conceptual organization of our first-person experience of the world and ourselves as agents in it. Essentially, the creation and use of compressive models by agents, such as biological recurrent neural networks, provide the structure to phenomenal experience. These models can be seen as the mathematical version of Platonic forms—the non-physical conceptual realities that capture the true essence of material reality (the shadows of forms) [29,43]. In this context, S stands for the descriptors of model characteristics and structure [28,44].

The central hypothesis in KT is as follows.

**Hypothesis 1**  (Structured Experience). *An agent has structured experience (S) to the extent it has access to encompassing, accurate, and compressive models to interact with the world. Program length and other characteristics deriving from the structure of the model being run determine the structure of experience [27,28]. The event of structured experience associated with presence—the sensation of perceiving oneself as being in a real place [45], or more broadly, what we may call ontophoria (the qualia of immersion in being itself, connoting an existential experience of presence within the entirety of reality), arises from the successful alignment between model-generated and external world data [31].*

The compressive nature of such models (programs) allows agents to store them (memory) and report them to the self and others, i.e., for the conscious (verbal, behavioral) report, which underpins current consciousness research. The qualitative aspects of structured experience are thus associated with the algorithmic features of the model used, including its length (models are programs in KT), which reflects the structure discovered in the data.

### 2.1. Emotions as Structured Valence

A crucial dimension of human experience is emotion and mood. Importantly, with regard to MDD diagnostic criteria, the Diagnostic Statistical Manual 5 (DSM-5) refers to the subjective concepts of mood and pleasure. In the agent model framework, we add *valence*, the output of the Objective Function. In cognitive neuroscience and psychology, valence is typically defined as the intrinsic attractiveness (positive valence) or adverseness (negative valence) of an event, object, or situation. It is an integral part of the emotional experience, representing one of the two primary dimensions of emotion—the other being arousal [46]. Valence is closely linked to the experiences of pleasure and pain.

It follows that our tenet represents *emotion or mood* as structured experience with valence, or *structured valence* for short. In other words, the experiential nuance of emotions is derived from the world model’s features together with a valence value produced by the agent’s evaluation of the model using its Objective Function. Valence here encompasses the spectrum from pleasure to pain. Whether valence is positive or negative, or only more or less positive (or negative), is not a crucial distinction in this framework. All that matters is that it is a scalar in some interval in R.

**Definition 2**  (Emotional state or Mood of an Agent). *The emotional state of the Agent is the tuple E = (Model, Valence). In first-person language, emotion is structured experience with valence, and can be described along dimensions characterizing model structure (simplicity, breadth, accuracy, etc.) and valence.*

The concept of emotion as a “world model with valence” can be seen as a synthesis of several key theories in the fields of psychology and neuroscience. First, appraisal theory posits that emotions arise from our cognitive evaluations of events, suggesting that our emotions are tied to our interpretation or understanding of the world around us [47,48]. This aligns with the “world model with valence” aspect of the agent model. Second, the valence component is consistent with Schachter and Singer’s two-factor theory [49], which emphasizes the role of cognitive interpretation in labeling our physiological arousal as a particular emotion [50]. Third, Barrett’s theory of constructed emotions further underscores the importance of cognitive processes, suggesting that our world model actively constructs emotions based on experiences, culture, and context [51,52]. Fourth, the functional approach to understanding emotions, as defined by Adolphs and Anderson, proposes that emotions should be understood by their functions, which can be seen as the changes and behaviors incited by our brain’s interpretation of the world [53]. This also resonates with the idea of emotion as a “world model with valence” in the agent framework. Finally, the agent perspective is well aligned with constructivist approaches [49], because world models in KT are hierarchical mathematical structures that map into biological circuits. As defined in this paper, these theories collectively provide a foundation for understanding emotion as a “world model with valence”.

### 2.2. Algorithmic Agent in MDD

The following formulation defines depression as an emergent property of a dysregulated system with persistently low valence.

**Definition 3**  (Depressed Agent). *Depression is a pathological state in which the output value of the Objective Function (valence) of an agent is persistently low.*

How does an agent reach a condition of persistently low valence? A key advantage of the abstract agent framework is that it allows the dissecting of alternative routes to answer this question, including the dysfunction of any of its modules.

The onset of depression in the physiological agent can stem from different types of stressors, both acute and chronic. It may be caused by various interacting pathological mechanisms, including neurotransmitter imbalances, hormonal dysregulation, inflammation, neuroplasticity changes, genetic susceptibility, and complex interactions between different organs and systems [54]. Alternatively, depression may originate from a traumatic event, where a sudden influx of novel information alters the agent’s world model, leading to a rapid and significant decrease in valence. This “model-shock” can destabilize the agent dynamics, rendering it unable to predict or secure positive future states—further contributing to the onset and persistence of depression. If the agent cannot compensate for the reduced valence due to such dysfunctions, it becomes trapped in a persistently low-valence state, which can be understood as depression—an emergent dynamical property of a dysregulated agent system.

This perspective conceptualizes depression as a state of disrupted cognitive and emotional stasis, emphasizing the persistence of reduced valence that distinguishes clinical depression from transient dysphoria. From an evolutionary standpoint, the mechanisms underpinning this maladaptive state may have roots in the very processes that shape our cognitive faculties. Thus, depression is more than a transient response; it is a pathological state posing a threat to the stability of the agent’s internal equilibrium, making it critical to address not just the symptoms but the underlying regulatory dysfunctions that perpetuate the disorder [55].

Within this framework, we can identify different agent components where dysfunction or natural changes may result in persistent low output values from the Objective Function, i.e., potential causes or etiological routes and symptoms of depression in the agent (see Figure 2). The constituents of the agent and their dysfunctions in the context of MDD are articulated as follows.

#### 2.2.1. The Modeling Engine

The Modeling Engine is responsible for building, refining, and running the agent’s model of the “universe”, which includes both the external world (environment) and the agent itself. This generative model is used to predict future events and compare these predictions with actual incoming data.

The Modeling Engine’s failure in producing accurate world models—deriving from errors in model construction, dysfunctional plasticity, etc.—is a threat to tele/homeostasis (preservation of pattern) and thus leads to low valence. Erroneous representations of the world influence the evaluation of valence by the Objective Function and the output of action plans by the Planning Engine, cascading into further detrimental effects on valence from bad plans and actions.

The *Simulator*, which may be viewed as part of the Modeling Engine, unpacks models into simulated data for various agent components, including the Modeling Engine and its model *Updater*, the Objective Function, and the Planning Engine. It allows the agent to run models and simulate the world. A dysfunction within the Simulator will have catastrophic consequences for all these interconnected modules. Faulty simulations (or Updater errors) will prevent accurate model updates, leading to poor or maladaptive world models that distort reality. Furthermore, faulty simulations impair the agent’s ability to correctly evaluate valence and plan effective actions, reinforcing the negative cognitive loops that are central to MDD. Without accurate simulations, the agent’s decision-making and ability to adapt to its environment will degrade, worsening symptoms such as hopelessness, indecision, and emotional evaluation.

The *Comparator* is responsible for assessing the congruence between the model-generated data (predictions) and the actual incoming data, generating an error signal to alert the engine of modeling failures and which is used to refine the model. Again, this is closely related to the evaluation of Free Energy in the Active Inference framework (AIF) [39], but here the comparison is not weighted by goal-oriented priors [28]. A Comparator malfunction will impact the model Updater, leading the Modeling Engine to produce incorrect models. It will also affect the agent’s ability to maintain (first-person) ontophoria, which depends on an accurate comparison between model-generated data and sensory data. When the faulty Comparator produces low errors, the agent becomes overconfident in flawed models, avoiding necessary updates, leading to distorted Objective Function evaluation (valence) and poor planning. On the other hand, when the Comparator erroneously signals high errors, the agent loses confidence in its models, triggering excessive updates and disrupting structured experience. This can lead to derealization, where the world feels fragmented or unreal, impairing both decision-making and effective interaction with the environment. Valence evaluation, which also hinges on having confidence in a world model, will be biased negatively.

Finally, the Modeling Engine is influenced by other modules, such as the Objective Function and Planning Engine. Models are built with a focus on elements critical to valence evaluation, and model selection quantifies the cost of different types of errors on overall utility. Just as utility functions guide optimal threshold selection in classification tasks by prioritizing costly errors, the agent’s models may reflect a negative cognitive bias if errors that lower valence are excessively weighted.

#### 2.2.2. The Objective Function

The Objective Function is a key agent component in the context of KT and, crucially so, in the study of MDD. It maps a model—whether that selected by the agent in the present moment or posited in the process of planning—into a scalar quantity, which in KT is directly identified with valence. This metric evaluates the agent’s state in relation to its goals. For natural agents, valence assesses the probability of achieving tele/homeostasis, thereby providing the basis for the selection of actions of future value. In biological organisms, natural selection selects agents that “stay” as patterns, a process that shapes features of the agent, such as the Objective Function and Modeling Engine. In our theory, the Objective Function value is the quantity optimized by the Planning Engine (discussed below). The Objective Function, like other agent modules, may be realized as a distributed system [56,57], with subsystems possibly tasked with assessing positive or negative contributors to tele/homeostasis. The computation of present or future contributions to valence may be very complex as it entails running the world model and compressing the result into a single number—explicitly or implicitly [37]. In the case of the human brain, many distributed systems are likely involved in its calculation, from the basal ganglia to the neocortex, as we discuss below. Recent investigations in depression using causal mapping methods are converging on dichotomous circuits reflective of symptomatic dimensions of positive and negative affect, which can be analogously mapped onto the Objective Function’s architecture dedicated to valence computation [58,59,60,61]. Furthermore, the computation of valence involves varying temporal scales, some of which are probably short (to enable fast reaction times) while others may require longer ones (e.g., rumination through the simulation of potential futures). The Objective Function thus may integrate evaluations across these disparate temporal domains.

By definition, the agent will act in any way it can to increase its Objective Function value, which may include steps for *exploration* and model building vs. *exploitation*. These trade-offs will result from the specific formulation of the Objective Function, e.g., the relative value of *delayed returns*. This refers to the idea that actions taken by an agent affect not only the immediate reward but also the future rewards. This critical aspect of reinforcement learning allows the agent to plan and make decisions considering long-term outcomes rather than immediate gratification. The agent interacts with an environment over discrete time steps to maximize its cumulative reward. At each time step *t*, it selects an action $a_{t}$ from a set of possible actions A, based on its current state $s_{t}$, and receives a reward $r_{t}$ from the environment. The environment then transitions to a new state $s_{t+1}$. The agent’s objective is to learn a policy π that maximizes the expected cumulative reward, also known as the return $O_{t}$, which is defined as $O_{t}=\sum_{k=0}^{\infty }\gamma^{k}r_{t+k+1}$, where γ∈[0,1] is a discount factor that determines the present value.

The mathematical notion of future value links intuitively with the experience of *anxiety*, an emotion that typically arises in response to perceived threats, potential harm, or negative future events. Thus, anxiety can be seen as a low valence state in anticipation of negative outcomes or a devaluation of expected benefits.

A dysfunctional Objective Function is reflected in maladaptive valence evaluation. Specifically, this pertains to a scenario where the function persistently yields low valence values, irrespective of the informational substrates presented for evaluation (including simulated scenarios from planning future actions). The complexity inherent in the appraisal of valence implies that elements such as the Simulator can be the potential loci of dysfunction. Furthermore, since world conditions can naturally lead to persistently low Objective Function values, the Objective Function must be sufficiently malleable to ensure the adaptation of the agent into new, harsher conditions. This highlights the importance of the plasticity of the Objective Function—valence recalibration. If plasticity is absent, an agent may not be able to recover following world insults. By *recalibration*, we mean a mechanism in the Objective Function to reset its output slowly to a baseline value if the world model (Objective Function input) does not change favorably after some period of time. For example, a persistently low value of valence due to past events may be detrimental in the long term since this state will focus the agent’s efforts on raising it even if this is actually impossible. A similar case can be made for persistent positive valence. In the literature, this is known as *hedonic adaptation*, the idea that people tend to return to a relatively stable level of happiness despite positive or negative events in their lives [62,63,64]. The *hedonic set point* hypothesis or this theory postulates that each individual has a baseline level of happiness or well-being to which they tend to return.

#### 2.2.3. The Planning/Action Engine

The Planning/Action Engine uses the current world model, Simulator, and Objective Function to formulate plans, which are then translated into actions at the external interface. For brevity, we merge planning and action generation, acknowledging their conceptual distinction. The function of the Planning Engine is to produce plans (and consequent actions) that increase the value of the Objective Function output (valence). Indirectly, planning also subserves modeling via exploration and experimentation, but this is a consequence of Objective Function maximization and the regulator theorem.

The Planning/Action engine of a depressed agent may produce plans that do not increase valence or that further decrease it (i.e., producing actions that impact the world with negative consequences for the agent). This potentially aligns with the “cognitive biotype” (see Section 3) of depression [12,65], suggesting an intricate interplay between planning mechanisms and depressive phenomenology.

Depressed patients often report a sensation of being “stuck”, unable to envisage an emotional state of higher valence. This can be attributed to two failure modes of the Planning Engine: first, an inability to identify a “target world” of higher valence, precluding the formulation of actionable plans. Mathematically, this could be expressed as a failure in both the local and global optimization of the valence landscape. Second, if such a target is identified, the Planning Engine may be unable to formulate plans to reach an identified more favorable world state with reasonable probability, i.e., a misestimation of the likelihood of reaching that state with the best found plan. Both scenarios induce a state of *hopelessness*, effectively paralyzing the agent into inaction. Although not explicitly mentioned as part of the definition of depression, this concept links with the notion of *existential despair*: a deep-seated sense of meaninglessness or futility, often accompanied by the inability to envision a future where circumstances improve [66].

#### 2.2.4. The World

The world, or environment, represents the all-encompassing environment where the agents operate, providing input signals (information) to which the world models are compared and the agents’ actions affect.

We refer here to an objectively hostile world in situations where the agent is subjected to inputs signaling legitimate threats to its equilibrium/stasis, suggesting a universally challenging environment that could diminish valence in a typical, healthy agent. Such conditions, e.g., the loss of a family member, often initiate depressive episodes. Yet, in resilient agents, episodes of diminished valence triggered by objectively adverse circumstances are transient, with recovery ensuing via the Objective Function’s adaptive recalibration. This plasticity may be absent in individuals exhibiting pathological responses.

### 2.3. The Agent Model and Dynamics

To better understand and treat MDD, integrating a dynamical systems approach with algorithmic and neurobiological perspectives is essential. The agent is a computational construct. But because computation is a dynamical process [67,68]—the Turing machine itself is a discrete dynamical system characterized by state transitions over time—the properties of the agent can be explored by viewing it as a dynamical system. From this point of view, models and other agent components are programs that are “run” with agent structure mapping directly onto dynamical system properties [28,44].

The connection between the agent model and dynamical systems theory provides powerful tools for analysis, including bifurcation theory, differential geometry, and topology. Analyzing the brain’s state dynamics and attractor landscapes offers more profound insights into the mechanisms underlying neuropsychiatric phenomena, such as multistability, critical transitions, and symptom emergence in conditions like depression, schizophrenia, and epilepsy. By integrating these dynamical tools, this approach bridges algorithmic features, brain dynamics, and clinical phenomena, offering novel perspectives for theoretical and empirical research in neuropsychiatry. This integration reveals the complex interactions and feedback mechanisms underlying depressive states, aiding in the development of effective therapies.

Along these lines, we recently introduced a framework integrating concepts from dynamical systems, computational modeling, and complexity to explore the relationship between neural dynamics, brain connectivity, and plasticity—all of them associated with the realization of the agent model in the biological brain [69]. This interdisciplinary framework can be used to analyze the mechanisms behind pathological disorders such as MDD and the potential therapeutic effects of psychedelics.

#### Landscape Erosion in MDD

Analysis of the dynamical trajectories associated with the neural state of the agent provides a different view of the interaction of the different agent modules. To understand MDD under this framework, we can map state coordinates to an extra dimension encoding the state of the Objective Function (valence) (in the same way that energy appears in classical dynamical systems). The generalization of this is a Lyapunov function [70], a function always increasing with time in a closed system except at equilibrium. In a normal, healthy brain, subjected to external inputs, the value of the Objective Function will fluctuate around a mean as trajectories explore the dynamical landscape (this corresponds to *free energy* in the FEP). In this sense, we may think of the external world as providing some steady state noisy input to the dynamical system until some significant changes occur (e.g., trauma or psychotherapy). Consistent with neuroimaging studies exploring healthy brain function, the landscape exhibits sufficient stability to allow for meaningful visits to substates while simultaneously possessing enough flexibility to avoid being trapped in a singular substate. In the brain affected by MDD, the dynamics are such that valence consistently hovers around a low value, akin to a trough in a landscape. In terms of dynamics, this is described as the system’s trajectories being anchored to an attractor within a low-valence region. To illustrate how this happens, we can think of depression as being triggered by a traumatic life event—an external input—that subsequently shifts the brain into these low-valence states.

Traumatic incidents reshape the dynamic landscape: synapses undergo modifications, serving as memory imprints of these events. Such transformations can create a pronounced attractor region, capturing trajectories and compelling the agent’s plans and actions toward escape. In a healthy agent, balancing mechanisms are at play: the system is geared towards eventual adaptation, finding a new equilibrium with valence levels rising again, even if the original cause of the valence drop remains. We propose that in a resilient brain, the Objective Function undergoes plastic recalibration, causing the pronounced trough to gradually level out. In the unhealthy brain, however, the landscape does not heal, possibly due to the pathological plasticity function [71]. Healing entails finding plans to act on the world to fix an external cause, adjust world models, or re-calibrate the Objective Function. For example, there is evidence indicating that Objective Function recalibration may happen during REM sleep [72], which may be impaired in sleep abnormalities (REM fragmentation). All these actions can change the dynamical landscape. On the contrary, in the pathological case and due to what we may call maladaptive plasticity [73], the recurrence of trajectories in this deep well may further deform it into a deeper minimum, and the trajectories may remain forever trapped. Inspired by the CANAL framework, we call this *landscape erosion* or canalization [73].

## 3. Translational Framework

Here, we present the first preliminary mapping of the agent model onto neurobiological substrates and biotypes. This translation is grounded in our current understanding of the brain region and network functions, as well as emerging data-driven biotype classifications [10,11,12,65] which, while promising, require further validation. While this framework will inevitably evolve as knowledge advances, it serves as an initial step toward demonstrating the model’s potential to bridge computational theory with neurobiological complexity. See Appendix Table A1 and Table A2 for details on each region and Appendix A.1 for a list of acronyms. Appendix C provides a more in-depth review of the relevant brain regions.

### 3.1. Circuits, Functional Networks and Biotypes in MDD

Given the different ways some key terms are used in the literature—namely circuits or pathways, (typically, “circuit” is used in the context of studying a function or behavior, while”pathway” is used in the context of studying signal transmission), functional networks, and biotypes—we begin by defining them as they will be used in this paper. Since our work employs a network neuroscience perspective, we start from the mathematical notion of network: a *network* consists of a set of interconnected elements called *nodes*, together with the set of their relationships (connections), represented by *edges*. More formally, a network is defined as a graph G=(V,E), where *V* is a set of nodes and *E* is a set of edges, each connecting a pair of nodes. From this foundation, we define the concepts of *circuits* (see Box 1) and *functional networks*.

**Definition 4**  (Brain circuit or pathway). *A brain circuit or pathway is an information processing network consisting of brain regions (nodes) exchanging information through structural connections (edges) including synapses, gap junctions, or ephaptic coupling, among others.*

**Definition 5**  (Functional Network). *A brain functional network is a network derived from the activity of a brain circuit where nodes are brain regions and edges capture a mathematical relationship between node activities.*

Box 1 **Brain circuits involved in MDD.**

  The *cortico-thalamic-striatal-cortical loop* (CTSC) is central to cognitive control, integrating motor, cognitive, and emotional information, and its disruption is pivotal in the pathophysiology of MDD [74]. Key regions within this loop include the orbitofrontal cortex (OFC) and dorsolateral prefrontal cortex (dlPFC), which are critical for decision-making, emotional regulation, and executive function. In MDD, dysfunction in these regions undermines the agent’s ability to evaluate and execute plans effectively. The thalamus (TH), serving as a relay for sensory and motor signals, exhibits altered activity and connectivity in MDD, leading to disrupted sensory processing and sleep disturbances. The striatum, essential for reward processing and motivation, often malfunctions in MDD, manifesting as anhedonia and a diminished drive. Components of the basal ganglia (BG), such as the globus pallidus (GP) and subthalamic nucleus (STN), contribute to the psychomotor retardation characteristic of depression. Additionally, dysfunction in the dopaminergic pathways of substantia nigra, crucial for mood and motivation, precipitates core symptoms of anhedonia and the lack of pleasure.

 The *mesolimbic dopaminergic pathway* (MLP) is crucial for reward processing, originating in the Ventral Tegmental Area (VTA), which projects dopamine signals to the nucleus accumbens (NAc), essential for feeling pleasure and motivation [75,76]. Dysfunction in this system can lead to anhedonia [77]. Key structures include the VTA, AMY, and NAc, which collaborate to evaluate the rewarding potential of behaviors. Changes in dopamine levels within this pathway are critical in depressive behaviors [78]. The NAc, part of the ventral (medial) striatum (VS), is central to processing positive reward, desire, and even the placebo effect. At the same time, the AMY plays a significant role in fear and threat detection. Due to its role in cognitive and emotional processing, the MLP is also implicated in addiction [79].

 The *prefrontal cortex and amygdala pathway* (PFCA) highlights the critical role of disrupted connectivity between the prefrontal cortex (PFC) and AMY in the pathophysiology of MDD and anxiety. Decreased functional connectivity between the AMY and the left rostral PFC has been identified in medication-naive MDD patients when processing fearful stimuli [80]. Neuroimaging reveals abnormally elevated metabolic activity in the AMY and orbital cortex in depression, with AMY activity positively correlating with depressive symptoms [81,82]. Functional disconnection between limbic and frontal regions, particularly between the AMY and the OFC bilaterally, and among the AMY, ACC, and PFC in the right hemisphere, has been demonstrated in MDD patients [83]. These findings suggest that an impaired modulation of AMY activity by the PFC may underlie aberrant emotional processing in depression and anxiety. Within the agent framework, the PFCA represents a critical intermodule circuit, integrating cognitive control and emotional regulation.

In our definition of circuits, an important aspect is their implicit causal and mechanistic role in information transmission and information processing—which is not necessarily the case in the definition of functional networks. However, the direct measurement of brain circuits can be challenging due to their intricate, small-scale nature and the limitations of non-invasive techniques. Functional networks derived from neuroimaging data serve as valuable proxies, allowing researchers to infer the functional connectivity and interactions between different brain regions and providing insights into the underlying neural mechanisms. Functional networks are studied using diverse neuroimaging or electrophysiological techniques, and they emerge from neural dynamics constrained by the structural connectome during spontaneous brain activity, or in response to stimulus or task-evoked perturbations [84,85,86]. One of the main challenges in neuroscience is to understand how functional networks arise from brain circuits. This is broadly described as the structure–function relationship of brain networks.

With these definitions in place, we discuss the notion of MDD biotypes. MDD is diagnosed when a patient manifests at least five out of nine potential depressive symptoms. This diagnostic criterion accommodates several hundred unique combinations of alterations in mood, appetite, sleep, energy, cognitive function, and motor activity. This impressive diversity underscores the prevailing consensus that depression is not a singular entity but exists in various subtypes. However, the neurobiological foundations—the biotypes—of these distinct depression subtypes’ manifestations remain largely unknown.

**Definition 6.**  *A biotype is a subtype of MDD characterized by specific neurobiological traits shared by a distinct subgroup of individuals with MDD behavioral and clinical symptoms that distinguish them from other individuals with MDD.*

We conceptualize the etiology of MDD at two levels of description: (i) the *agent type* or *algotype* and (ii) the *biotype*. Biotypes give rise to symptoms and functional network alterations that can be used to cluster subjects in what we call *functional network (FN) biotypes* (Box 2), following the work of Williams et al. [10,11,12]. Algotypes are useful to analyze the role of module disruptions as theoretical etiological routes to low valence, but they need not map directly to human MDD biotypes (which may involve more widespread disruptions). Biotypes, in turn, give rise to patterns of functional co-activations among brain regions reflected in FN biotypes. FN biotypes are often derived from intrinsic and task-evoked functional activity in healthy and clinical populations [12].

Box 2 **Functional networks involved in MDD.**

  The *Default Mode Network (DMN)*: A network of brain regions that are active during rest and introspection; it is involved in self-referential thinking, autobiographical memory, and mental time travel [56,87]. Abnormal activity and connectivity within the DMN have been associated with MDD, contributing to the altered processing of self-related information and the negative self-referential thinking—or **rumination**—seen in depression.

  The *Cognitive Control Network (CCN)*: Also known as the Central Executive Network, it plays a crucial role in goal-directed behavior, attention, and working memory. It comprises regions like the lateral prefrontal cortex, the posterior parietal cortex (PPC), the FEF, part of the dorsomedial prefrontal cortex (dmPFC), the dlPFC, and the anterior cingulate cortex (ACC) [88]. Unlike the DMN, the CCN is highly active during cognitive tasks and contributes to the “task-positive network”, which also includes the middle temporal region, supplementary motor cortex, and the fronto-insular operculum, showing robust task-related activation [89]. In MDD, altered interactions between the DMN and CCN impact cognitive control and emotion regulation [90], potentially leading to cognitive discontrol and deficits in emotion regulation.

  The *Salience Network (SN)*: This primarily includes the fronto-insular cortex, dorsal anterior cingulate cortex (dACC), AMY, and temporal poles; it detects and responds to salient stimuli such as acute stress [91]. It is essential for paralimbic emotional processing and plays a significant role in emotional control through its extensive subcortical connectivity. Notably, the SN is critical in facilitating the switch between the DMN and the CCN [92]. Abnormalities in connectivity and activity within the SN, particularly in the anterior insula and dACC, are observed in MDD and may underlie biased attention toward negative information and diminished responsiveness to positive stimuli (anhedonia).

  The *Attention Network (AN)*: It consists of the Dorsal Attention Network (DAN) and the Ventral Attention Network (VAN). The Dorsal Attention Network mediates goal-directed, top–down attention, enabling individuals to focus on tasks and filter out distractions. It primarily involves brain regions such as the intraparietal sulcus (IPS) and the frontal eye fields (FEFs) [88]. The VAN is involved in bottom–up, stimulus-driven attention, which allows the brain to reorient attention toward salient or unexpected stimuli. It includes regions such as the temporoparietal junction (TPJ) and the ventral frontal cortex (VFC) [88,93]. Disruptions in the AN, particularly in the balance between the DAN and VAN, can lead to difficulties in maintaining attention and an increased susceptibility to distractions, contributing to the cognitive deficits in MDD [88,93,94].

  The *Negative Affect Network (NAN)*: This involves regions such as the AMY, anterior insula, medial prefrontal cortex (mPFC), and the ACC, crucial for processing and regulating negative emotions. Overactivity or dysregulation of this network can lead to persistent negative mood states and a negative bias, which is common in depression. This results in a heightened sensitivity to negative stimuli and an impaired ability to regulate negative emotions, contributing to the symptoms of anxiety and depression [12,95,96].

  The *Positive Affect Network (PAN)*: This includes regions such as the ventral striatum, OFC, ventromedial prefrontal cortex (vmPFC), and the ACC, essential for processing positive emotions and rewards. Dysfunction in this network can lead to anhedonia. The PAN is critical for the experience and anticipation of reward, motivation, and positive reinforcement, and its disruption can severely affect mood and behavior [12,97,98,99].

In this work, we explore how the current systems in neuroscience supports the idea of a set of depression biotypes that correspond to distinct alterations in circuits and functional networks. Then, informed by the literature on brain circuits and functional networks (Box 1 and Box 2), we investigate how circuits, functional networks, and biotypes are integrated into the agent framework to deepen our mechanistic understanding of the various manifestations of MDD.

Recent work has shown that disconnections within task-free salience and DMN circuital elements map onto symptoms such as anxious avoidance, loss of pleasure, threat dysregulation, negative emotional biases, and poorer daily functioning [100]. However, limited evidence supports the hypothesized one-to-one mappings between clinical circuit scores and specific phenotypes, challenging common assumptions about neural–phenotype relationships. The findings suggest that a simple one-to-one mapping between circuits of interest and specific symptoms and behaviors is not supported within current samples and theoretical frameworks [10,11,12].

An exception is the recently confirmed cognitive biotype of depression, representing about a quarter of depressed patients and characterized by prominent impairments in the cognitive control domain such as executive function and response inhibition [65]. This biotype is associated with reduced activation in the DLPFC and dACC within the cognitive control circuit, suggesting a distinct neural mechanism underlying this biotype. This reduced prefrontal activation aligns with the cognitive control biotype proposed by Williams et al. based on neuroimaging findings and the role of these regions in cognitive control. Similarly, Drysdale et al. identified four neurophysiological subtypes of depression defined by distinct patterns of dysfunctional connectivity in limbic and frontostriatal networks using fMRI in a large multisite sample [101]. These subtypes further support the heterogeneity of depression and underscore the need for personalized approaches based on neural connectivity patterns.

### 3.2. From Agent Modules to MDD Functional Biotypes Through Circuits

The diversity in the manifestations of MDD, both in etiology and symptomatology, underscores the complexity of its underlying mechanisms. We propose that situating neurobiological circuits within the agent framework and its modules may help elucidate the mechanisms behind the different MDD FN biotypes, keeping in mind the caveat that the agent elements are abstract and may be realized in a highly distributed, hierarchical fashion in the brain [102]. By aligning disruptions in agent modules with biological circuits, we aim to understand how these perturbations lead to various manifestations of MDD, such as rumination, anhedonia, and cognitive dysfunction.

Models, simulation, and planning systems are closely and hierarchically integrated into the brain across different brain structures and temporal scales (fast vs. slow learning [103] and fast and slow simulation [104,105]). In our exploration, we focus on the principal actors, revealing the critical roles and interactions that underpin the brain’s capacity for modeling, simulation, and planning. We provide an overview of this section in Table 1.

#### 3.2.1. The Modeling Engine

**Figure 3:**
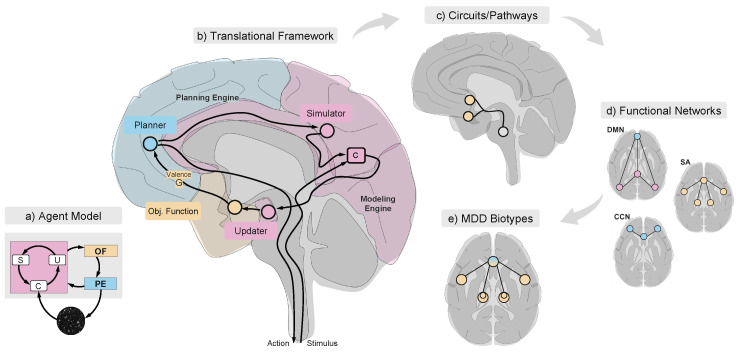
From the agent model to brain circuits, functional networks, and biotypes. To highlight the goals of the proposed framework, we provide a tentative map or agent modules to brain regions, circuits, and candidate biotypes based on the current literature. Proposed mapping of agent model and agent elements (**a**) to high-level brain circuits (**b**)—Modeling Engine in pink, Planning Engine in blue, and Objective Function (valence evaluation) in yellow. The high-level circuital model maps into structural/anatomical circuits (**c**) and, finally, into observed functional networks that can be derived from fMRI (**d**). Features from these networks can be used as biomarkers for the definition of patient clusters or FN biotypes (**e**) [10,11,12,65,100]. The characteristics of each biotype (alterations in the activity of regions or their connectivity) will reflect alterations of the main agent modules involved. As an example, we display a particular FN biotype (${NS}_{A+}P_{A+}$ in [12]), with altered activity in the Objective Function (AMY, BG, and ACC). Most FN biotypes present alterations in more than one agent module.

The Modeling Engine is responsible for building the agent’s internal model of the external “world” and generating predictions about future events, which can be compared with actual incoming data from the sensory interfaces. In our framework, the Modeling Engine of the human brain is linked with several key anatomical regions, including the hippocampus, the cerebellum, and selected areas within the frontal cortex. These regions are not only crucial for their individual functions but are also interconnected to form complex circuits that underpin the engine’s capability (Figure 3).

As mentioned, KT proposes that ontophoria emerges from accurate comparisons of model predictions and data. In the agent model, this occurs at the Comparator, where prediction errors are evaluated by comparing the Modeling Engine’s predictions to incoming data. The canonical cortical microcircuit model provides the neurobiological basis for the Comparator, enabling a hierarchical comparison of sensory data with model-generated predictions through converging feedback and feed-forward streams [106,107]. There is growing evidence that layer V cortical pyramidal cells play a key role in this process, serving as the convergence point for sensory data and priors (predictions), particularly in posterior cortical regions [39,108,109,110,111].

This view integrates the Global Workspace Theory, the Free Energy Principle, and the dendritic integration theory (DIT), emphasizing the importance of thalamo-cortical and cortico-cortical loops in consciousness [110,112,113]. The Comparator’s role in generating structured experience is further supported by the “posterior hot zone” hypothesis, where research shows that disruptions in these processes can significantly impact conscious states [114,115,116,117]. In MDD, a malfunctioning Comparator can reinforce flawed models (leading to overconfidence in negative beliefs and poor decision-making) or cause excessive updates, derealization and loss of structured experience, and lower valence. These disruptions can fuel the negative feedback loops typical of depression, contributing to feelings of hopelessness, disconnection, and impaired planning.

##### Modeling Engine Dysfunction

The brain regions associated with the Modeling Engine are part of larger circuits essential for cognitive and emotional processes (Box 3). The PCC plays a pivotal role within these networks, forming connections with the hippocampal system [118]. Specifically, the PCC acts as a gateway, transmitting spatial and related information from the parietal cortex to the HC and facilitating the flow of object information from the temporal lobe to and from the HC via the perirhinal cortex [119]. Additionally, the PCC and the OFC provide the hippocampal system with routes for reward-related and emotional information, enhancing the complexity of episodic memory formation [120]. Outputs from the HC primarily project to the entorhinal cortex (EC), which is located in the medial temporal lobe and is crucial for memory, navigation, and time perception. The EC serves as a major conduit, linking the HC with various cortical and subcortical regions [121]. Strong functional connectivity between the PCC and lateral OFC suggests an integration of reward and punishment information within this network, with the parahippocampal region showing activation during negative or sad mood inductions [122]. In healthy individuals, the PCC responds to happy and neutral stimuli, but in MDD, increased functional connectivity with the lateral OFC biases PCC inputs toward negative or sad events. Significant circuit changes are also observed in the lateral OFC, which shows increased functional connectivity with the PCu and the AG [123]. The enhanced connectivity with the PCu, involved in the sense of self and agency [120], and the AG, associated with language, correlates with the negative self-perception and low self-esteem seen in depression. These functional connectivity differences are linked to the severity of depression symptoms and are less pronounced in patients undergoing antidepressant treatment [123]. Furthermore, by integrating information related to reward, punishment, self-perception, and language, the regions presented within this module are integral to the CSTC loop.

Box 3 **Brain regions associated with the Modeling Engine.**

*Angular Gyrus* (AG): A region of the parietal cortex showing structural changes such as reduced volume or cortical thickness in MDD patients. In MDD, regional homogeneity in AG decreases but appears to increase after treatment with electroconvulsive therapy [124]. As part of the DMN, the AG supports semantic processing and connects perception, attention, spatial cognition, and action, as well as recalling episodic memory—all functions disrupted in MDD [125]. Neurophysiological subtypes of depression, characterized by functional connectivity between the right AG and DMN, may predict treatment outcomes [126]. Within the agent framework, the AG as part of the Modeling Engine, integrates sensory information and aids in semantic processing, contributing to accurate world modeling and effective planning. Its dysfunction leads to distorted world models, impairing decision-making and homeostasis.

  *Posterior Cingulate Cortex* (PCC): It is crucial for regulating emotional and memory-related processes [127] and plays a key role in internally directed cognition [87]. It is involved in episodic memory tasks, such as autobiographical memory and future imagining, as well as spatial navigation and scene processing [128,129]. The PCC exhibits high connectivity and metabolic activation levels [87], making its activity a key neuroimaging biomarker for depression symptoms and treatment outcomes [130]. Moreover, increased functional connectivity of the PCC is linked to rumination in depression [131]. As part of the Modeling Engine, the PCC integrates self-referential and emotional information to update the world model. Impairment of the PCC affects emotional processing and a coherent sense of self.

  *Precuneus* (PCu): A region in the medial parietal cortex integral to self-referential processing, spatial navigation, and episodic memory [132]. It is anatomically connected to the OFC, facilitating the integration of cognitive and emotional information. In MDD, abnormal activity in the PCu can impair self-awareness and memory. From the algorithmic agent perspective, the PCu belongs to both the Modeling and Planning Engines. It helps to maintain an accurate model state by integrating spatial and autobiographical data, which are essential for self-awareness.

  *Hippocampus* (HC): An evolutionarily conserved structure, it is central to spatial memory and cognitive mapping across species [133]. Cells are placed in the HC encode locations, forming cognitive maps or internal representations of the world, which are essential for navigation and decision-making across different cognitive domains [121,134], from navigation to mathematics [135]. The HC plays a key role in representing latent, multidimensional spaces where various aspects of information are encoded [103,136,137,138,139]. Crucially, the HC regulates prefrontal cortical function, and disruptions here can impair concentration and memory common in MDD [140]. Volumetric imaging studies show that factors like early and family history and burden of illness contribute significantly to decreased HC volumes in MDD patients, compared to healthy controls [141,142,143]. As part of the brain’s Modeling Engine, the HC builds cognitive maps, and its impairment leads to distorted environmental modeling and cognitive deficits in depression.

  *Cerebellum (CB)*: It is an evolutionarily ancient structure with uniform histology, supporting broad processing functions without distinct areal boundaries [144]. It coordinates voluntary movements and maintains balance, integrating sensory inputs with motor commands. It also modulates cognition and emotion through the *universal cerebellar transform*, maintaining behavioral homeostasis [145]. Recent insights suggest that the CB might employ fast ephaptic communication [146,147], facilitated by its densely packed folds, for rapid processing. The spatial organization of somatosensory, cognitive, and affective representations within the CB relies on polysynaptic connections with functionally distinct cortical regions [148], which fMRI studies have mapped [149]. In MDD, studies have found a significantly smaller CB and vermis—an area responsible for regulating emotion [150,151], correlating emotional dysregulation and anxiety [152]. In the agent framework, the CB functions as a fast, forward modeling/planning system [105,153], integrating internal models to control motor actions and supporting a range of neurological functions through its extensive interactions with other brain structures [104].

Dysfunction within the Modeling Engine can potentially arise from abnormalities in any of these brain regions, leading to disturbances in functional networks such as the DMN. Such disturbances are associated with specific FN biotypes, each characterized by unique symptoms. For example, disruptions in the DMN are common in MDD patients exhibiting rumination symptoms. Abnormal hippocampal activity within the Modeling Engine may impair planning processes necessary for generating adaptive behaviors that enhance affective valence, resulting in persistent rumination and manifesting as hyperactivity within the DMN [11]. Moreover, regions of the Modeling Engine are also part of the AN. Dysfunctions in these areas can affect the agent’s ability to maintain attention and shift focus appropriately. According to Williams et al. (2017) [11], general hypoconnectivity within the AN suggests an additional *inattention* type, clinically expressed as hypovigilance and loss of alertness, leading to false alarm errors. This hypoconnectivity can impair the agent’s capacity to stay focused and alert, contributing to cognitive deficits observed in MDD. The interplay between the DMN and AN disruptions can thus contribute to a broader range of cognitive and emotional symptoms.

#### 3.2.2. The Objective Function

The Objective Function computes valence based on the agent’s world model, integrating external and internal sensory data. This computation is crucial for formulating action plans and may involve various brain regions, including the sensory cortices, insula, ACC, and other areas involved in interoceptive awareness and cognitive processing. Insofar as some actions may be taken independently of others (e.g., initiating motor actions to reach for a bowl of fruit and regulating core body temperature), valence may be evaluated by subsystems independently before integration into a global valence assessment.

##### Objective Function Dysfunction

The brain areas involved in the Objective Function form interconnected circuits crucial for emotional regulation, reward processing, and stress response (Box 4 and Box 5).

As a part of the CTSC, the sgACC and PFC are connected with the TH and AMY, forming a feedback loop that influences mood and emotional regulation. The PVT acts as a relay center, integrating inputs from the AMY and conveying them to cortical areas like the sgACC and PFC, which process and regulate emotional and cognitive responses. This loop also involves the NAc, which receives dopaminergic inputs from the VTA and integrates signals from the PFC and AMY, modulating reward and aversion processes.

Box 4 **Brain regions associated with the Objective Function (1 of 2).**

  *Hypothalamus* (HYP): This is central to systemic homeostasis maintenance, regulating key physiological imperatives such as hunger, thirst, and thermoregulation through its various nuclei. The lateral hypothalamic area initiates hunger, while the ventromedial hypothalamus signals satiety [154]. The HYP integrates physiological signals to influence valence evaluation. It modulates inputs from PFC and NAc, contributing to a holistic assessment of internal states and environmental demands [155]. In MDD, hypothalamic dysfunction disrupts circadian rhythms, appetite, and stress responses, generating persistent negative valence signals [156]. Chronic activation of the HPA axis elevates cortisol levels and heightens the stress response, promoting threat overestimation and avoidance behaviors.

  *Thalamus* (TH): Known as the “Relay station”, it transmits sensory and motor signals to the cerebral cortex and modulates consciousness and alertness. Its role extends to the pathophysiology of MDD, where it is implicated in mood and emotion dysregulation [157]. The central role of thalamocortical oscillations is particularly noteworthy. Increased thalamic excitation can disrupt these rhythms through its extensive projection to the neocortex—a phenomenon observed in MDD patients. This disruption can lead to symptoms such as insomnia, anhedonia, and cognitive impairment [158]. The *Paraventricular Thalamus* (PVT)—a specific nucleus within the TH, is involved in regulating stress responses and negative emotional behaviors, with connections to key brain regions implicated in mood disorders [159]. Its dysfunction contributes to the emergence of symptoms such as anhedonia.

  *Nucleus accumbens* (NAc): It is associated with positive reinforcement and the processing of rewarding stimuli. “Pleasure” or “liking” is generated by a small set of “hedonic hot spots” within the limbic circuitry with a particular implication of the NAc [3,160]. However, research has shown that the NAc also plays a role in aversive conditioning and the processing of negative stimuli [161,162,163]. In a healthy brain, the NAc is crucial for motivation, reward processing, motor function, and learning, especially for the anticipation and experience of reward (positive valence), which facilitates goal-directed behavior and reinforces learning through positive outcomes. Individuals with MDD produce reduced responses to rewarding outcomes in the NAc, particularly during the consummatory phase of reward processing, and are associated with anhedonic symptoms [2]. Additionally, reduced activity in the NAc can lead to decreased motivation and goal-directed behavior [164,165]. Increasing evidence supports the NAc’s modulatory role in the pathogenesis of MDD, making it a promising therapeutic target [166,167,168].

The OFC corticostriatal circuits are critical for behaviors such as evaluation, affect regulation, and reward-based decision-making. Dysfunction in these circuits is linked to psychiatric disorders like MDD. Therapeutic neurostimulatory techniques such as DBS, ECT, rTMS, and tDCS can rectify abnormal OFC corticostriatal activity, improving clinical symptoms [169].

Functionally, the PVT plays a significant role in emotional processing and conditioned behaviors through its connections with the HYP, PFC, and AMY [170]. Patients with psychotic MDD exhibit decreased functional connectivity between the HYP and sgACC, associated with cortisol dysregulation and symptom severity [171]. MDD patients also display reduced intrinsic connectivity between the AMY and regions involved in emotional processing [172] and between the NAc and regions associated with reward processing, executive function, and the DMN [162,173]. These alterations in NAc connectivity mediate the association between MDD and *anhedonia* [165] (a symptom defining one of the FN biotypes in [11,12]), impairing the Objective Function evaluation of positive contributions to valence. Consistent findings of striatal hypoactivation in the reward circuit suggest an additional type characterized clinically by “anhedonia” and a loss of sensitivity to reward stimuli [11]. Anhedonia, *apprehension*, and *threat dysregulation* have been linked with changes in connectivity and activation of the SN, PAN, and NAN [11]. In MDD, disruptions in these functional networks can lead to impaired reward processing and heightened negative emotional responses.

Box 5 **Brain regions associated with the Objective Function (2 of 2).**

  *Amygdala* (AMY): It is central to emotion processing, including both positive and negative rewards [174,175,176]. Research indicates distinct neural circuits for positive and negative valence within the AMY [177]. It is particularly involved in fear and anxiety and plays a critical role in emotional memory and the stress response [178,179,180]. In a healthy brain, the AMY evaluates the emotional significance of stimuli, especially those related to threats, generating appropriate behavioral and physiological responses (negative valence) [181], while in MDD, AMY hyperactivity contributes to a negative bias in emotional processing, and negative stimuli are perceived more intensely [182]. This heightened amygdala activity is linked to increased stress and anxiety, worsening depressive symptoms [183]. In the agent framework, AMY hyperactivity in MDD leads to overestimating threats and underestimating rewards, distorting valence computation in the Objective Function.

  *Subgenual anterior cingulate cortex* (sgACC): Known to regulate emotional behavior and autonomic responses, it is also closely connected with AMY, hippocampus, and NAc. Findings from Fox et al. [184] show that fMRI functional connectivity differences in the sgACC were linked to the efficacy of TMS sites in the dorsolateral PFC for treating MDD, underscoring the sgACC’s potential as a target for antidepressant interventions. This insight led to identifying optimal TMS target coordinates and inspired approaches using tDCS to engage the broader cortical network associated with the sgACC-seed rs-fcMRI [22]. More recent work found that sgACC overactivation increases heart rate, elevates cortisol levels, and exaggerates responsiveness to threat, mirroring the stress-related symptoms of depression and anxiety [185]. In MDD, hyperactivity in the sgACC can lead to a distorted evaluation of emotional stimuli, resulting in a negative bias and an overestimation of negative outcomes. As a result, inaccurate valence signals are sent to the Planning Engine, leading to ineffective or maladaptive planning and decision-making processes.

  *Orbitofrontal cortex* (OFC): It is a key integrative hub linking sensory inputs across modalities—gustatory, olfactory, somatosensory, auditory, and visual—with reward processing and decision-making. It has reciprocal connections with several brain structures, including AMY, ACC, insula/operculum, HYP, HC, areas in the BG including VS and dorsal striatum, periaqueductal gray, and dlPFC [169,186]. Functionally, the OFC evaluates the affective value of stimuli (valence), guides behavior based on reward predictions, and modulates emotional responses [187]. Its structural connections enable determining homeostatic stimulus value [169,174,188]. The OFC acts as an integrative center where positive and negative valence signals combine to compute a comprehensive assessment of *valence* [186], where it synthesizes input from regions like the nucleus accumbens (NAc) and AMY. In MDD, the OFC’s function is significantly impaired, leading to anhedonia and disrupted decision-making due to altered reward evaluation and processing [82,186]. Additionally, the OFC coordinates with the HC to update models and contrast them with data for model update [136], contributing to the Planning Engine by using refined models to guide decision-making and action selection. This dual role is crucial for maintaining accurate cognitive maps and effective behavioral strategies.

#### 3.2.3. The Planning Engine

The Planning Engine is closely connected to the Objective Function, and it should also be represented in a related, distributed, hierarchical manner. At lower hierarchical levels, it includes classical homeostatic regulatory systems such as the hypothalamus (HYP). Higher up, the cerebellum (CB) contributes to coordinating and timing precise, rapid movements and cognitive functions, aiding in planning and predicting outcomes of movements and behaviors [145]. At the highest level, planning is partly associated with executive function, and, in particular, the PFC, from MC, to the inferior frontal cortex, mPFC, ACC, OFC, and dlPFC.

##### Planning Engine Dysfunction

The brain areas involved in the Planning Engine (Box 6) are interconnected through the CTSC intermodule circuit. The HYP communicates with the prefrontal cortex regions (dlPFC, mPFC, and OFC) via pathways that integrate autonomic and endocrine responses with cognitive processes, influencing the agent’s ability to plan and execute goal-directed behavior. The dlPFC is connected to the mPFC and OFC through extensive cortical networks that facilitate the integration of executive functions, working memory, and emotional regulation. These prefrontal regions receive input from the TH—which acts as a relay station—and project to the striatum. The PCu integrates these cognitive and emotional processes by connecting with the mPFC and dlPFC, playing a role in self-referential thinking and the Default Mode Network. Dysregulation within this loop leads to impaired planning and maladaptive decision-making. The HYP’s disrupted signaling affects stress responses and homeostasis, while the dlPFC’s hypoactivity and the mPFC’s hyperactivity contribute to cognitive control deficits and negative emotional biases. The OFC’s role in evaluating rewards and punishments becomes skewed, leading to poor decision-making and anhedonia. The PCu’s involvement in self-referential thoughts can exacerbate rumination and negative self-perception. Within the PFCA intermodule circuit, the PFC interacts closely with the AMY (see Objective Function section) to integrate cognitive control with emotional regulation. This connectivity allows the PFC to modulate emotional responses generated by the AMY, ensuring that plans and actions are both logically sound and emotionally appropriate (i.e., increase valence). MDD disrupts the critical connectivity with the AMY and sgACC that is necessary for effective cognitive control and emotional regulation potentially associated with future contributors to valence [178,179,189,190,191]. This leads to impaired top–down control over emotional responses, dysfunctional decision-making, and maladaptive behaviors.

Box 6 **Brain regions associated with the Planning Engine.**

  *Dorsolateral prefrontal cortex (dlPFC):* It is the highest hierarchical level (“meta”) for planning and executive functions in the human brain, playing a crucial role in complex cognitive processes such as decision-making, problem-solving, and the regulation of goal-directed behavior, thereby distinguishing humans from other primates [192]. It is involved in cognitive control, working memory, and selective attention and is significant in MDD, where dysregulation potentially disrupts goal-directed cognition [10,193]. Research suggests an imbalance between left and right dlPFC activity in MDD, with left dlPFC hypoactivity associated with negative emotional judgment and right dlPFC hyperactivity linked to attentional modulation [96,194] and cognitive bias associated with greater and more sustained AMY reactivity [96]. This functional asymmetry may have a structural basis, as MDD patients show lower structural asymmetry in the dlPFC compared to healthy controls [195]. Cognitive control dysfunction contributes to MDD psychopathology and emotion dysregulation [196]. The tDCS of the dlPFC has shown promise in improving cognitive function and reducing depressive symptoms [196]. In MDD, dlPFC dysregulation impairs planning and decision-making, leading to indecisiveness, reduced concentration, and difficulty in goal-setting. The imbalance between the left hypoactivity and right hyperactivity skews emotional processing and cognitive control, resulting in maladaptive planning and ineffective strategies for managing negative emotions and stress.

  *Medial PFC* (mPFC): It plays a key role in various memory-related functions, particularly in autobiographical and emotionally charged memories. Its connectivity with the HC positions the mPFC as important for memory consolidation and retrieval [197]. Beyond memory, the mPFC is integral to goal-directed behaviors, including planning and decision-making, by evaluating the potential rewards of different choices and adapting actions based on these valuations. It is involved in performance monitoring, identifying when outcomes fall short of expectations, and generating predictions about future outcomes by integrating past experiences with the current context [197]. This anticipatory function enables individuals to foresee the possible consequences of their actions and to modify their behavior to align with desired outcomes. In MDD, disruptions in mPFC functions lead to impaired planning, maladaptive decision-making, and a persistent negative outlook.

  *OFC* and *PCu*: By using refined models to guide decision-making and select appropriate actions, the OFC ensures accurate cognitive maps and effective behavioral strategies. The PCu aids in generating and evaluating action plans using past experiences and spatial contexts. Dysfunction in the PCu can lead to ineffective planning, mirroring the cognitive impairments seen in depression.

  *HYP:* Although associated with the Objective Function due to its role in evaluating low-level valence contributors, the HYP also controls these variables (e.g., thermoregulation), illustrating the “onion-layer” hierarchical structure of the brain. Dysregulation of the HYP in MDD leads to continuous negative feedback to the Planning Engine regarding the physiological state, resulting in maladaptive decision-making that prioritizes the avoidance of perceived threats over pursuing rewarding activities. Chronic stress signals cause the agent to avoid social interactions and previously pleasurable activities, reinforcing depressive symptoms.

  Other areas in the agent’s Planning Engine include regions and circuits associated with acting on the world. The *motor cortex (MC)* and, in particular, the *supplementary motor area (SMA)*, play a crucial role in orchestrating voluntary movements, coordinating information from various parts of the brain to execute complex motor actions. Abnormalities in the SMA have been associated with motor deficits in MDD patients [198,199]. The *brainstem (BST)* regulates basic life functions like heart rate, breathing, and sleep, which are vital for maintaining the physical capability to act on the world. It also houses the reticular formation, which is involved in arousal and consciousness. The BST innervates the Hypothalamic–Pituitary–Adrenal (HPA) axis and frontolimbic circuits, which are dysfunctional in MDD.

The *Corticospinal Tract* (CST) is essential for voluntary movements, and its abnormalities in depression may relate to reduced energy levels and overall depression severity [200,201]. Functional connectivity studies have shown increased connectivity between the left SMA and motor-related regions but decreased connectivity with the BG during high-frequency finger-tapping tasks in MDD patients [198]. Motor retardation in MDD is associated with unbalanced motor control, evidenced by altered cerebral blood flow and motor activity levels in regions such as the cingulate cortex and SMA [202]. These findings suggest that MDD involves disruptions in both structural and functional connectivity of the SMA, contributing to motor symptoms and cognitive deficits. Diffusion tensor imaging has revealed decreased white matter integrity in the right solitary tract, connecting the BST to the AMY (Objective Function), in MDD patients, together with volumetric reductions in the BST [203,204]. These findings indicate that structural and functional alterations in brainstem regions and their connections to other brain areas contribute to MDD pathophysiology.

We propose that malfunctions in the agent’s Planning Engine can result from disruptions in key brain areas and circuits, altering associated functional networks such as the DMN, CCN, and AN. These disruptions correspond to distinct FN biotypes, each with unique symptoms. For example, agents with Planning Engine dysfunction may fail to generate plans that enhance valence, leading to textitrumination and anxiety, possibly due to alterations in the DMN-associated mPFC. Alternatively, disruptions in CCN-related areas like the dlPFC may impair impulse regulation, causing the selection of short-term valence-enhancing but long-term detrimental plans, indicative of the *cognitive dyscontrol* FN biotype.

Moreover, regions of the Modeling Engine are also part of the AN, and their dysfunction can affect the agent’s ability to maintain attention and shift focus appropriately, contributing to the inattention biotype linked to hypoconnectivity within the AN, as noted by Williams et al. (2017) [11]. This hypoconnectivity can impair both the Planning and Modeling Engines by reducing the agent’s capacity to stay focused and alert.

#### 3.2.4. The World

In the context of MDD, the world, or the environment, significantly influences both the onset and progression of the disorder by transmitting inputs to and receiving outputs from the agent. Biologically, the world’s inputs are processed via the sensory organs and integrated by different brain regions to form a comprehensive perception of the external environment or the agent’s body. Stressful life events, childhood trauma, and ongoing adversity increase the risk of developing MDD, representing negative inputs that disrupt the agent’s equilibrium and hinder the maintenance of an optimal Objective Function output. A healthy agent typically formulates effective strategies to interact with the world and sustain a satisfactory level of valence. However, an unhealthy agent might struggle to develop and implement such strategies or fail to recalibrate and adapt to changes in the world, as seen in cognitive subtypes of MDD [65], where executive function (planning) is impaired. This impairment can lead to further deterioration of world conditions and, consequently, more adverse inputs, perpetuating the cycle of depression.

In MDD, the interaction between the agent and the world is often maladaptive, characterized by impaired sensory processing and motor responses, leading to a cycle of negative feedback. Sensory inputs may be filtered through a negative bias, and motor outputs may reflect decreased motivation and physical activity or result in more adverse world conditions, further enhancing depressive symptoms. In this line, the world represents an opportunity for MDD intervention, acting as a gateway to “reprogram” the agent. Beyond psychotherapy, which impacts the agent through the world interface, helping the agent cultivate more adaptive methods to interpret (model and evaluate) and respond (plan) to the world’s inputs, effective treatments often require changes to the patient’s environment or their ways of engagement (behavioral therapy). Furthermore, lifestyle adjustments such as regular physical activity, a healthier diet, and improved sleep hygiene can boost the agent’s interaction with the world, optimizing the chances for higher valence as computed by the Objective Function.

## 4. Relation to Other Theories, Empirical Support, and Predictions

### 4.1. Relation to Other Computational Theories of MDD

The algorithmic agent framework is an overarching paradigm that integrates various computational psychiatry approaches. By conceptualizing mental health through agents optimizing for valence via modeling and planning interactions with their environments, this framework encompasses models focused on reward processing, decision-making, and learning—key aspects in computational psychiatry.

#### 4.1.1. The Free Energy Principle and Active Inference

In previous work [28,205], we highlighted the conceptual compatibility between the agent framework and Karl Friston’s Free Energy Principle and Active Inference (FEP/AIF) [113,206]. Both frameworks emphasize hierarchical processing, predictive coding, and the maintenance of adaptive neural models for healthy cognitive function [113,205,206]. For instance, the evaluation of prediction errors, critical in FEP, aligns with the error evaluation at the Comparator in the algorithmic agent model.

FEP/AIF provides a causal statistical framework accounting for action, perception, and learning by positing that an adaptive agent minimizes free energy (or *surprise*, represented as the negative log-likelihood of the data given the model, J=−logP(y)) to maintain stasis and resist disorder. This minimization involves maximizing the evidence for the agent’s internal generative model of sensory data. The minimization of surprise is a dual process: it involves finding models that not only match incoming data but also remain consistent with prior models, which encode the agent’s homeostatic goals and a statistical notion of complexity as a departure from prior models. This balances statistical complexity (related to algorithmic complexity) and accuracy. The agent seeks to minimize surprise by adjusting its internal models to better explain sensory inputs while aligning with its homeostatic priors. This partly parallels KT, where the agent aims to minimize the description length of its internal models by balancing model complexity and data fit. However, in KT, the primary goal is the optimization of the Objective Function, which may rely partly on the simplicity of the found models as defined through the Kolmogorov complexity.

Our work in [205] further clarifies the relationship between FEP/AIF and KT by deriving principles of probability and Bayesian inference from the algorithmic agent model in AIT. This derivation shows that FEP/AIF can be viewed as a specific realization of the agent model in a Bayesian context, where probabilistic reasoning emerges as a strategy for the agent to manage uncertainty and optimize its interaction with the environment. Finally, in FEP/AIF, the agent seeks not only to compress sensory data using Bayesian methods but also do so in alignment with intrinsic goals encoded as priors—a specific proposal for the realization of the Objective Function in the brain.

In FEP/AIF, psychiatric disorders like depression are primarily understood as failures in this adaptive process [207,208,209,210,211,212,213], where maladaptive internal models give rise to persistent negative predictions and behaviors. In KT, maladaptive models can lead to low valence, but other routes—such as dysfunctions in the Objective Function or Planning Engine—are also considered.

#### 4.1.2. Reinforcement Learning and Other Perspectives

Reinforcement learning (RL) and predictive coding (PC) are computational frameworks naturally encompassed within the KT algorithmic agent model. RL involves agents learning optimal actions through trial and error, guided by rewards and punishments, aiming to maximize cumulative reward. It emphasizes the evaluation of actions based on their expected outcomes and the refinement of policies through experience [214]. PC (subsumed by FEP/AIF and KT), on the other hand, focuses on minimizing prediction errors by continuously updating the agent’s internal model to better match the sensory input, viewing the brain as a predictive machine, where discrepancies between predictions and actual observations drive model updates [206]. In the KT model, both RL and PC are integrated into a unified process, where the agent optimizes its internal models, compresses information, and adapts its behavior by balancing rewards, prediction errors, and model complexity.

Recent research has explored the relationship between depression and RL, revealing significant alterations in RL processes among individuals with depression. Studies have found that depression is associated with impaired reward prediction error signals, decreased reward sensitivity, and altered learning parameters [215]. These RL disruptions have been linked to depression symptoms and may predict treatment outcomes [216]. Neuroimaging studies have shown that symptom improvement following cognitive behavioral therapy is associated with the normalization of learning parameters [216]. Furthermore, research has demonstrated that individuals with depression exhibit context-dependent learning impairments, particularly in negative contexts, suggesting a negativity bias in learning rates [217]. Additionally, Huys et al. (2015) [218] link depression to low expected rates of net utility (valence), arising from maladaptive priors that distort internal and external state construction. This mirrors the KT framework, where the agent’s faulty predictive models lead to persistently negative expectations about the future, reducing the valuation of potential outcomes. Both models highlight that depression involves biased predictions and decision-making, where the expectation of future utility is diminished due to maladaptive internal models. Huys and Maia [26] categorize computational models in psychiatry into synthetic, algorithmic, and optimal models. Synthetic models are biophysically detailed, bridging neural dynamics with behavior. Algorithmic models, such as RL, focus on simplified, statistically validated constructs. Optimal models use Bayesian frameworks to evaluate decision-making against theoretical optima. This categorization aligns with the KT framework, which integrates synthetic modeling for neural circuit dynamics and algorithmic approaches for predictive coding, Objective Function evaluation, and planning in depression. Similarly, Maia and Frank [219] use RL models to explore dopamine function in cortico-basal ganglia-thalamo-cortical (CBGTC) circuits, emphasizing interactions between the basal ganglia (BG) and cortex in an actor–critic framework. This parallels the KT model’s distinction between planning, modeling, and Objective Function evaluation while delving deeper into dynamics and plasticity in depression. Khalegi et al. [220] further classify computational psychiatry models into phenotypic, neurobiological, and intermediate models. Whole-brain models based on neural mass models (NMMs) fit into the neurobiological category, providing detailed system-level insights into how neural alterations manifest as psychiatric symptoms. Bennet [221] and Nelson [222] discuss computational cognitive models in depression, focusing on decision-making, reward processing, and predictive coding. Disruptions in these cognitive functions, such as negative bias and impaired reward learning, reflect the KT framework’s focus on altered modeling and Objective Function evaluation. Charlton [223] explores how ketamine rapidly alleviates suicidal ideation by modulating synaptic plasticity and neural circuitry involved in mood regulation. The use of RL models to understand value-based decision-making in suicide risk reflects the KT framework’s emphasis on neural plasticity and predictive modeling in affective disturbances.

### 4.2. Testable Predictions

Theories closely related to KT, such as FEP and AIF, or RL, offer testable predictions about brain function and behavior. Central to the FEP/AIF framework is the idea that the brain minimizes prediction errors by continuously updating its internal models to better match sensory input. This leads to predictions about sensory processing, testable through tasks that manipulate prediction errors, such as oddball paradigms where unexpected stimuli elicit measurable neural responses like mismatch negativity [206,224]. In the context of decision-making, AIF predicts that individuals select actions minimizing expected free energy, which can be experimentally tested using behavioral tasks under uncertainty, coupled with neuroimaging to track brain regions involved in belief updating and action selection [113,225]. Furthermore, PC and AIF predict that psychiatric disorders like depression or schizophrenia involve disruptions in hierarchical prediction error minimization. This can be examined using reinforcement learning tasks assessing maladaptive reward processing and prediction error sensitivity [207,226]. AIF’s emphasis on allostatic regulation and uncertainty minimization also provides predictions regarding the role of bodily states in cognitive processing, testable through tasks that manipulate interoception and assess its influence on decision-making and affective states [206,226]. We provide a more in-depth comparison of KT and AIF in Appendix D.

The KT framework also predicts that agents will select the simplest models available, all else being equal [31]. This compression principle suggests testable hypotheses about the brain favoring simpler world models, which can be investigated through tasks manipulating model complexity and observing behavioral and neural responses. Neuroimaging studies could examine whether the brain shows preferential activation for simpler representations during inference or prediction tasks. As a computational neurophenomenology theory, KT posits that model structure—in terms of dynamical and geometric/topological features—shapes experience and emotion. This could be empirically tested by analyzing the geometric and topological properties of neural activity patterns and correlating them with subjective reports of emotion. For example, investigating how changes in the brain’s state space (e.g., dimensionality, topology, or curvature) correspond to different emotional states using techniques like manifold learning or persistent homology [28].

Emotion is a crucial dimension of both first-person experience and behavior. To validate the formulation of emotion within KT, researchers can derive a standard emotion labeling system by establishing qualia structures across participants based on similarity relationships [227] or by creating neural embedding frameworks that map human emotions into vector space representations, confirming a shared “qualia structure” across individuals [228,229]. Demonstrating that participants with matching qualia structures exhibit similar neural data associated with emotional states, as deduced from the geometry and topology of trajectories in phase space, would support this aspect of the theory [69].

In the context of MDD, the KT agent framework predicts that dysfunctions in specific brain circuits involved in model-building or planning will lead to behaviors and symptoms consistent with incorrect models or impaired executive functions. This mapping can be tested using neuroimaging alongside cognitive and affective assessments to investigate the relationship between neural circuit dysfunctions and MDD symptomatology. For example, disruptions in circuits critical for planning may be associated with increased rumination or impaired decision-making.

Regarding treatment efficacy, the KT framework suggests interventions targeting specific dysfunctional circuits or processes should alleviate corresponding symptoms. Testing these predictions involves evaluating the effectiveness of targeted therapies and observing associated changes in neural activity and cognitive function, which we discuss in the next section.

## 5. Treating the Depressed Agent

The treatment of MDD involves a multifaceted approach, including pharmacology, brain stimulation, psychotherapy, and lifestyle adjustments. By analyzing these interventions through the lens of the algorithmic agent model, we gain deeper insights into how each treatment modality alters brain dynamics and influences recovery. Table A3 links brain regions with interventions affecting them.

Within this framework, all treatments—pharmacological, brain stimulation, psychotherapy, or lifestyle interventions—can be viewed as mechanisms that temporarily reshape the brain’s dynamical landscape. Pharmacological interventions, such as antidepressants and psychedelics, modify the structural parameters governing brain dynamics. Psychedelics, for instance, act on serotonin receptors, potentially “flattening” the landscape and facilitating novel neural trajectories, as seen in frameworks like REBUS [112], CANAL [73], and Neural geometrodynamics [69]. This flattening increases the brain’s capacity for reconfiguration, allowing a transient escape from maladaptive states [69,230,231]. The process has been likened to “annealing” in physical systems, allowing the brain to escape local minima and explore new configurations [232]. Furthermore, psychedelics enhance metaplasticity, opening periods of increased brain plasticity that support this process [69,112,233].

The notion of pathological canalization [234] provides further insight into disease progression. When the system becomes dynamically trapped in a local minimum, a first phase we may call dynamical canalization (Type A canalization) takes place, with associated depressed mood (low valence). At this stage, external stimuli like social support, therapeutic interventions, or transient landscape flattening via psychedelics may enable escape from the local minimum. Alternatively, a second phase involving plasticity loss or cell damage (Type B canalization) may occur, further entrenching depressive states. Combined and repeated therapies may be necessary to facilitate both transient escape and gradual landscape reshaping [234].

### 5.1. Brain Stimulation

Brain stimulation therapies apply electrical or magnetic stimulation to specific brain regions to modulate neural activity and improve the functionality of agent modules.

For instance, Electroconvulsive therapy (ECT) involves the application of electrical currents to the brain that induce a controlled seizure [235]. It is one of the most effective treatments for severe depression, particularly when patients are experiencing suicidal ideation or psychosis. Recent work supports the hypothesis that the evoked seizure is the key to efficacy [236]. In the canalization paradigm, it can be interpreted as a strong external forcing on the state that can dislodge neural dynamics from the trapped attractor.

On the other hand, deep brain stimulation (DBS) requires surgical implantation of electrodes into specific subcortical brain regions, such as the sgACC or NAc [166,237]. DBS electrodes deliver sustained electrical stimulation, modulating the neural activity of the targeted brain structures. Targeting the sgACC or NAc with DBS can be interpreted as modulating the Objective Function of the algorithmic agent.

At the non-invasive level, transcranial magnetic stimulation (TMS) uses electric fields, generated by time-varying magnetic fields, to stimulate targeted regions of the brain, and in particular the left dlPFC [184]. TMS is non-invasive and has fewer side effects than ECT. Furthermore, transcranial direct current stimulation (tDCS) is another non-invasive technique that uses a low, constant current to stimulate specific brain areas [238,239]. tDCS has shown promise in MDD (anodal tDCS of the left dlPFC in MDD without drug resistance [14,240]), but results across different montages and studies warrant further larger scale controlled studies. The current is delivered through scalp electrodes and typically targets the left dlPFC (similarly in TMS, transcranial magnetic stimulation). Connectivity between dlPFC and subgenual cingulate cortex (SGN) suggests that modulating the activity of the dlPFC may have downstream effects on mood regulation and emotional processing through its connections with these limbic areas [184]. Targeting the dlPFC with TMS or tDCS can be seen as an intervention that primarily affects the Planning Engine and, indirectly, the Objective Function (since the evaluation of valence needs to consider future rewards).

### 5.2. Psychotherapy

Psychotherapy addresses the cognitive, emotional, and behavioral aspects of depression [241], by modulating brain dynamics and altering synaptic connectivity. From the KT perspective, psychotherapy reprograms the agent by influencing core modules of the brain’s cognitive architecture. While each type of therapy has unique methods, many overlap in the brain systems they target and their mechanisms of action. *Cognitive Behavioral Therapy (CBT)* and *Mindfulness-Based Cognitive Therapy (MBCT)*, frequently used for MDD, both engage the Modeling Engine. CBT corrects cognitive distortions and restructures negative thought patterns [242], while MBCT reduces rumination by increasing present-moment awareness [243]. Together, they improve the Objective Function by promoting balanced self-evaluation and emotional stability. Therapies such as *Interpersonal Therapy (IPT)* and *Dialectical Behavior Therapy (DBT)* also play a significant role in MDD management by targeting social dynamics and emotional regulation. IPT enhances the Modeling and Planning Engines by improving representations of social interactions and refining social behavior [244]. DBT combines acceptance and change strategies to strengthen emotional regulation and interpersonal effectiveness [245]. *Psychodynamic Therapy* and *Eye Movement Desensitization and Reprocessing (EMDR)* have been applied in the context of MDD to reprocess maladaptive memories and internal conflicts. Psychodynamic therapy resolves internal struggles by bringing unconscious patterns to conscious awareness [246], while EMDR re-integrates traumatic memories into a more adaptive framework, easing emotional distress [247]. In contrast, *Acceptance and Commitment Therapy (ACT)* focuses on optimizing the Planning Engine in MDD patients by promoting value-based actions and acceptance of internal experiences [248].

### 5.3. Lifestyle and Alternative Treatments

Lifestyle interventions, such as social relations [249], exercise [250,251,252], sleep hygiene [253], and dietary changes [254], have shown to play a role in treating MDD. These interventions aim to improve overall physical and mental health, leading to environmental changes that positively impact the Objective Function. Furthermore, healthier habits may induce neurobiological changes that promote accurate world models (Model Engine) and improve emotional processing (Objective Function) via enhanced plasticity.

### 5.4. Neuropharmacology

#### 5.4.1. Antidepressant Treatment

Pharmacological treatments for MDD primarily target the monoaminergic neurotransmitter systems, including serotonin, norepinephrine, and dopamine. The rationale for targeting these neurotransmitter systems stems from the monoamine hypothesis of depression, which suggests that a deficiency in monoamine neurotransmitters contributes to the development of depressive symptoms [54]. Common classes of antidepressants include selective serotonin reuptake inhibitors (SSRIs) and serotonin–norepinephrine reuptake inhibitors (SNRIs), which modulate the neurobiological circuits implicated in MDD to correct dysfunctional neural activity.

#### 5.4.2. Psychedelic Treatment

Psychoactive neuroplastogens like psilocybin and LSD modulate brain dynamics by acting as agonists or partial agonists of serotonin 5-hydroxytryptamine (5-HT) 2A receptors [16,73,112,255], which are abundant on the apical dendrites of neocortical pyramidal cells in Layer V. Desynchronization of ongoing oscillatory rhythms in the cortex may trigger the subjective psychedelics effects [256], likely initiated by 5-HT2A receptor-mediated excitation of deep pyramidal cells. For instance, when psilocybin binds to these receptors, it increases the excitability of pyramidal neurons, depolarizing them and making them more susceptible to incoming inputs [257]. This effect can lead to changes in the firing patterns of these neurons and alterations in the overall neural activity.

By flattening the neural dynamical landscape, psychoactive neuroplastogens increase the entropy and complexity of neural dynamics, disrupting functional integration and promoting a more flexible neural state. This shift, amplified by the plasticity-enhancing effects of these substances, manifests as an acute systemic increase in disorder and a potentially longer-lasting increase in complexity affecting both short-term dynamics and long-term plastic processes. Such disruption enables the brain to escape maladaptive attractor states associated with MDD, facilitating the formation of new, more adaptive patterns of neural activity and connectivity [69,112].

During the acute phase of neuroplastogen action, there is a flattening of the modeling landscape [258] that can be described as a relaxation of priors [112]. The effects on 5-HT2AR can be interpreted as disrupting model inputs (priors) into the Comparator—where data and the model converge for the validation and generation of structured experience [28]. Increased errors reported by the Comparator force the brain to explore alternative models, facilitating the breakdown of maladaptive thought patterns. Enhanced neuroplasticity supports the long-term modification of the Modeling Engine, fostering new, healthier cognitive models and emotional responses [182,259].

Psychedelics also impact the Objective Function through their effect on key brain regions. In MDD patients, they modulate the AMY [260], increasing its response to emotional stimuli, which correlates with mood improvements. This heightened responsiveness may normalize emotional processing, enabling a fuller range of emotions that is often blunted in depression [182]. In healthy subjects, however, psychedelics typically reduce AMY reactivity to negative stimuli, potentially alleviating anxiety and fear [180,261]. Barret et al. (2020) [262] found that one-week post-psilocybin, negative affect, and AMY response to facial affect stimuli, were reduced, whereas positive affect and dorsal lateral prefrontal and medial OFC responses to emotionally conflicting stimuli were increased. Discrepancies in findings may be attributed to differences in healthy and clinical populations as well as experimental conditions.

Additionally, psychedelics significantly impact the BG, particularly within the VS/NAc. They modulate both dopaminergic and serotonergic systems, enhancing responsiveness to rewarding stimuli and alleviating anhedonia. Psychedelics increase the power of high-frequency oscillations and decrease their frequency in the NAc, while reducing low gamma oscillation power—effects mediated by 5HT2A receptors [263]. Additionally, they boost dopamine release in the NAc [264,265], leading to enhanced reward responsiveness during the acute phase of treatment. Psychedelics also promote long-term neuroplasticity in these regions, supporting sustained mood improvements by restructuring maladaptive neural pathways [259,266]. This is partly mediated by long-term changes in the Objective Function, promoting new behavior patterns and goal-setting conducive to mental health and well-being [112].

In the Planning Engine, psychedelics promote neuroplasticity, particularly within the PFC [259]. Activation of 5-HT2A receptors by psychedelics or serotonin triggers a robust glutamate-dependent increase in the activity of pyramidal neurons, preferentially those in layer V of the PFC [267]. This suggests that psychedelics are potent modulators of prefrontal networks through complex serotonin–glutamate interactions [267]. Although acute effects may impair executive function in healthy subjects [268], the long-term neuroplastic changes may enhance planning and decision-making.

Finally, the thalamus (TH), acting as a nexus of agent modules, plays a crucial role in mediating the effects of psychoactive neuroplastogens. We recall that the Cortico-Thalamo-Striatal-Cortical Loop (CTSC) is implicated in MDD and is crucial for integrating motor, cognitive, and emotional information. Its dysfunction is central to the pathophysiology of MDD. Psychedelics modulate thalamic activity, altering sensory experiences by affecting the thalamus’s role as a gateway for sensory information. Psychedelics lead to decreases in cerebral blood flow and BOLD signal in hub regions, such as the TH, ACC, and PCC [269]. This modulation alters directed connectivity within CTSC pathways in humans, suggesting that a disintegration of information processing within these loops is underlying the psychedelic state [270].

### 5.5. Combining tES with Psychedelics

While both tES and psychedelics have independently demonstrated significant therapeutic potential in treating depression; combining these treatments could potentially yield synergistic effects, surpassing the benefits observed when each is used independently. The combination is especially interesting from the point of view of plasticity. On the one hand, tES relies on the brain’s plasticity for therapeutic effects. tDCS induces long-term potentiation (LTP) or depression (LTD) of the glutamatergic system, which is N-methyl-D-aspartate (NMDA) receptor- and calcium-dependent [271]. On the other hand, psychedelics produce not only profound concurrent alterations of brain dynamics but also powerful after-effects with a window of increased neuroplasticity lasting at least up to one month [259].

The interaction of tDCS with pharmacological agents has been studied in numerous studies (see [272] for an overview) and, in particular, serotonergic drugs. Serotonin affects memory formation by modulating LTP and LTD. Accordingly, acute SSRI administration enhanced LTP-like plasticity induced by tDCS in humans. In pioneering studies, a single dose of the SSRI citalopram (CIT) enhanced both the amplitude and duration of the after-effects of anodal tDCS until the same evening of stimulation, and it reversed the excitability diminution seen after cathodal tDCS into facilitation [273]. Likewise, for paired associative stimulation (PAS), acute application of CIT enhanced LTP-like PAS-induced after-effects and abolished LTD-like PAS-induced after-effects [274]. Other studies have shown that chronic application of CIT increased and prolonged the LTP-like plasticity induced by anodal tDCS for over 24 h, and converted cathodal tDCS-induced LTD-like plasticity into facilitation [271]. More recently, it has been shown that acute serotonin enhancement modulates tDCS after-effects and has largely similar modulatory effects on motor cortex neuroplasticity regardless of the specific dosage [275]. Taken together, these experiments suggest that chronic serotonergic enhancement results in a strengthening of LTP-like glutamatergic plasticity, which might partially explain the therapeutic impact of SSRIs in depression and other neuropsychiatric diseases. This supports the approach of boosting the neuroplastic effects of anodal tDCS by serotonergic enhancement, a potential approach for the use of tDCS for the treatment of neuropsychiatric disorders.

## 6. Discussion

MDD is a complex disorder arising from various etiological routes, leading to variable symptoms and treatment responses. A deeper understanding of its etiology and associated synaptopathy [71] is essential for developing effective, individualized treatments that consider specific symptom profiles and underlying causes [8]. This requires a theoretical framework to guide measurement [7], research into etiology and patient subtypes, and treatment strategies [10,12,101]. Such a foundation can facilitate the development of generative, mechanistic models of the disorder at the biotype or individual level—what we refer to as *neurotwins* in reference to the notion of a digital twin, which is a virtual model designed with sufficient detail to accurately reflect a physical object or system, commonly used for simulation, analysis, and control purposes to optimize its operation.

Identifying agent elements in neurobiology can provide a blueprint for building models to explore the causes, stratification, and treatment of MDD. In this paper, we have proposed an initial draft of this map. Mapping the abstract agent model onto the complex “spaghetti code” of biological systems is a crucial step in this program. However, the hierarchical and intricately woven nature of neural networks—essential for managing complexity [205], optimizing performance, and ensuring stability [113,206,214,224,276,277,278]—adds significant complexity to this task.

Mechanistic models of complex systems, such as the brain, represent a significant advancement over empirical observations or phenomenological approaches. They provide a structured and predictive framework that enhances our understanding of underlying processes and interactions, crucial for scientific inquiry. Unlike empirical observations that document phenomena under certain conditions, mechanistic models explain why and how these occur, essential for fields like neuroscience and physics to predict and manipulate outcomes based on the system’s internal workings [279,280]. In neuroscience, such models allow for simulating and predicting neural behavior, offering insights into brain function and dysfunction and enabling the development of targeted therapeutic interventions—including brain stimulation and pharmacological treatments. Constructing these models often requires interdisciplinary collaboration, integrating insights from various fields [281,282].

With advancements in brain stimulation technologies, such as multichannel systems and focused ultrasound stimulation, the prospect of network-level interventions becomes feasible [283]. To maximize their potential, a constructivist approach is essential—mirroring engineering practices—since the parameter space of brain stimulation is too vast for exhaustive empirical exploration. By leveraging the agent framework, it is natural to approach the design of therapies through simulation grounded on mechanistic models.

Moreover, beyond recognizing distinct biotypes reflecting different etiological routes, personalized treatment can greatly benefit each patient. As part of the emerging field of computational neurotherapeutics, we and others are pursuing research on treating neurological conditions such as epilepsy and Alzheimer’s disease [107,258,284,285,286,287]. By developing personalized, mechanistic, whole-brain models that capture the intricacies of brain structure and function in health and disease, we can simulate the impacts of brain stimulation and pharmacological, such as psychedelics, and other interventions to deliver a paradigm shift in neurology [18,23,25,225,288].

**Figure 4:**
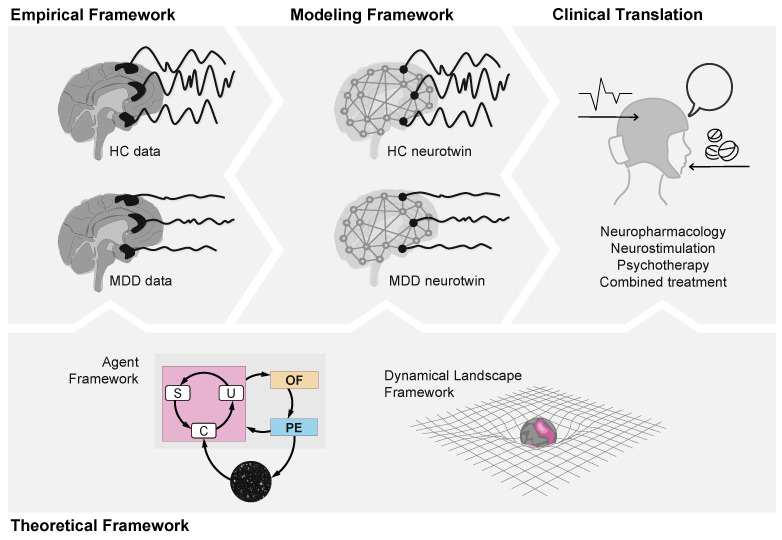
The agent and dynamical landscape frameworks provide a theoretical basis for the development and interpretation of data analysis methods and mechanistic computational models of the brain. This can be employed for a model-based design of therapeutic interventions such as pharmacology, brain stimulation, or even psychotherapy, as well as their combination. In the figure, we display the process of data assimilation from a group of reference healthy subjects/controls (HCs) and from MDD patients of a particular subtype. The resulting sets of models can guide the design of an intervention for the normalization of the dynamics and connectivity profile of the patients. The treatment can be designed at the group [289] or individual level.

Extending these innovations into psychiatry is the next frontier. The ultimate goal of this program is to create parsimonious, mechanistic generative models that elucidate sentient behavior and its pathologies [18], reshape current nosology in more biological dimensions, and provide advanced therapeutic interventions (see Figure 4). Achieving this requires interdisciplinary research in theoretical and applied aspects of brain modeling, clinical neuroscience, and psychiatry.

Whole-brain models aim to represent dynamical characteristics of neural function and derive measurable quantities such as electrophysiology or neuroimaging data, as well as represent the effects of interventions. Depending on the use case, more detailed mechanistic realism is needed. For example, in the case of epilepsy, it is important to represent mechanisms of generation and propagation of seizures in networks that brain stimulation and drugs can influence, as well as the effects of these interventions. Given the complexity of representing brain function, a multiscale approach is taken [281]. This is normally achieved by breaking down the system into mesoscale patches or volumes in a parcellation of the brain aligned with presumed function and connecting them with the connectome as a starting point. Current models use from some fifty to several thousand parcels. The activity of each parcel is modeled using neural mass models, which are in turn inspired or derived from microscale modeling [290]. Finally, hybrid models combine biophysical and physiological aspects of the brain [21,23].

Although ambitious, this program builds on existing research and technological advancements in computational power, numerical simulation, and AI. Computational models for psychedelics can currently explain their acute (but not long-term) effects, e.g., an increase in entropy [230,231,291]. While the concept of MDD whole-brain computational models has been discussed [25,292,293], no biologically inspired model of MDD is currently available.

Once a modeling framework is established, it can be used for personalization and treatment design. Typically, models are fitted to data by selecting specific features, such as functional connectivity ([294] and others). A particularly interesting approach that does not require the selection of features is to leverage large datasets to derive latent model descriptions for biotype identification [295,296] and for the definition of loss functions for data assimilation and treatment optimization. The basic idea is that it should be possible to leverage the low dimensionality intrinsic in neuroimaging data. An autoencoder trained to compress data can capture the low dimensional character of the data in its latent space (this is essentially hierarchical data compression). This can then be exploited for data fitting and clustering, e.g., identifying biotypes and differentiating healthy from pathological data, which can be used to design therapeutic outcomes via simulation [296].

Figure 4 illustrates some of the lines of work in the computational neuropsychiatry program described above. Variational autoencoders can be used to stratify patients and identify biotypes in the latent space. To personalize whole-brain computational models, the subject data can be projected into its latent space, where it can be compared to model-simulated data in a principled way. Semiology data can help start the personalization process from a biotype archetype of the model. Once the model is personalized, the effects of brain stimulation can be evaluated in silico. A latent space analysis can be useful to specify an optimization function, that is, where is the healthy region in the phase space that the stimulation should bring the dynamics to.

While a detailed discussion is beyond our scope here, key requirements for a computational modeling approach to MDD, aligned with the agent framework and combined treatment concepts, include the following:

- Exhaustive Brain Parcellation: Starting from a brain parcellation encompassing cortical and subcortical regions clinically relevant in MDD, covering all the agent modules.
- Parametrizable Circuits: Representing all potentially affected circuits in a way that allows parameters to characterize regional or connectivity alterations and inter-circuit coupling.
- Plasticity Mechanisms: Implementing mechanisms for plasticity (state-dependent and state-independent) and its dysfunction—synaptopathy.
- Effects of Intervention: Include representations of various therapeutic interventions—pharmacological treatments (e.g., neuroplastogens), brain stimulation techniques, psychotherapy, and lifestyle modifications—and model their acute and long-term effects on neural dynamics and plasticity.

Finally, the agent model within the Kolmogorov framework naturally extends to multi-agent simulations, allowing for the study of complex social interactions by instantiating multiple agents with unique algorithmic models. Agent-Based Social Simulation (ABSS) has emerged as a major tool for modeling such social systems, enabling researchers to study the emergent behaviors of individual agents and the resulting macro-level social phenomena [297,298,299,300]. This capacity enables the framework to analyze social dynamics and collective behaviors, akin to *psychohistory* in Asimov’s vision [301], or what we could call *Algorithmic Sociology*. Through these simulations, we can study how prevalent individual dysfunctions, such as MDD, impact social structures, decision-making, and collective well-being, providing insights into how mental health issues at the individual level ripple through societal interactions.

## 7. Conclusions

In this paper, we have tackled the challenge of defining depression and understanding its etiology by integrating algorithmic and neurobiological perspectives. We have argued that the abstract agent framework—which is closely linked to Karl Friston’s Active Inference/Free Energy Principle—can link to neurophenomenology (first-person experience and associated symptoms) while providing a blueprint to interpret the roles of brain circuits and dynamical phenomena. Although much remains to be carried out, these initial steps align with our ultimate goal: constructing a mechanistic model of brain function within a computational neuropsychiatric context [18], particularly focusing on depression.

We then reviewed current therapeutic approaches in light of the algorithmic agent model, exploring associated notions of agent types and biotypes and candidate pathways to depression. We emphasized the potential of combined therapies, including brain stimulation and psychedelics, highlighting the roles of neural dynamics and plasticity.

Finally, we described the process of creating digital twins to optimize treatment, with a special focus on the role of patient subtypes. Our work underscores the importance of personalized, data- and model-driven approaches in treating MDD and opens new avenues for integrating technology and neuroscience in mental health care.

## Figures and Tables

**Table 1:** Tentative mapping of different agent modules to specific brain regions, circuits, functional networks and associated symptoms or biotypes. Brain regions are listed in a descending spatial hierarchy (the symbol > means “contains”). Regions that are highlighted in bold represent those depicted in Figure 3, emphasizing their particular significance or involvement. This organizational structure helps to clarify the complex interactions and functional networks contributing to various symptoms and dysfunctions. See Appendix Table A2 for details on each region, and Table A3 for acronyms.

Agent Module Dysfunction	Brain Regions	Circuits	Functional Networks	FN Biotypes
Modeling Engine	AG; PCC; OFC; PCu; HC; CB	*Intramodule:* PCC ↔ HC; PCC ↔ PCu; OFC ↔ HC; *Intermodule:* CSTC loop (OFC, PCC, PCu);	DMN, AN	Rumination; Inattention
Objective Function	HYP, TH > PVT; BG > VS > NaC; AMY; SgACC; OFC	*Intramodule:* HYP ↔ PVT; TH ↔ PVT; AMY ↔ sgACC; AMY ↔ PVT; VTA ↔ NaC; MLP (VS, NaC, AMY); OFC ↔ AMY; OFC ↔ NAc; OFC ↔ HC; OFC ↔ dlPFC; OFC HYP; NAc *Intermodule*: CSTC loop (sgACC, PFC, TH, AMY, NaC, VTA, OFC); PFCA	SN, PAN, NAN	Anhedonia, Apprehension, Threat Dysregulation
Planning Engine	mPFC; dlPFC; mPFC > OFC > PCu; HYP	*Intermodule:* CSTC loop; PFCA; CST	DMN, CCN, AN	Rumination; Cognitive Dyscontrol; Inattention

**Figure A1:**
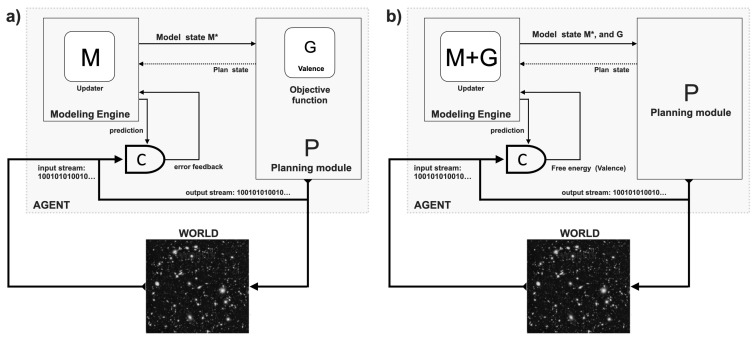
KT and AIF agent models and etiology of MDD. Schematic view of the agent models in KT and FEP/AIF. (**a**) In KT, the Objective Function is explicit, while in AIF, it is implicit in the loss function (free energy). (**b**) In AIF, the Planning Engine seeks to minimize free energy or surprise (negative valence). All the agent elements, in either case, can be affected and lead to depression in KT, i.e., persistent low valence: (i) Modeling Engine creates models leading to low valence; (ii) Objective Function returns low valence regardless of the models and displays no plasticity to “recalibrate”; (iii) Planning Engine delivers ineffective or detrimental plans for valence; and (iv) world provides inputs with an implicit stasis threat that cascades down to low valence. Further details: The agent interacts dynamically with its environment and is involved in a continuous exchange of data with the external world. The Modeling Engine runs the model and makes predictions of future data (both from external interfaces as well as the agent’s own actions) and then evaluates the prediction error (from the Comparator C) to update the model. The Simulator (not shown for simplicity) is a shared infrastructure used to run simulations for planning or valence evaluation. The Planning Engine (P) runs counterfactual simulations and selects plans for the next actions (agent outputs). The Updater receives as inputs prediction errors from the Comparator to improve the model. The other agent element is the Objective Function (which may also be plastic), which the agent aims to optimize through a choice of afferent actions selected by the Planning Engine.

**Figure A2:**
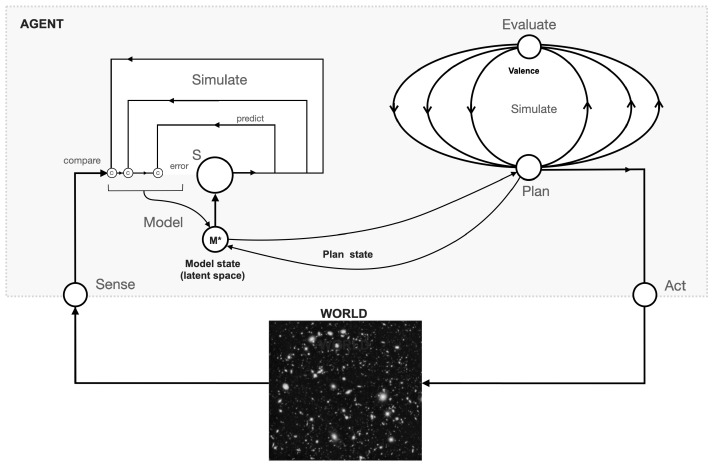
The hierarchical agent: Agent diagram displaying hierarchical processing of inputs (as in the predictive coding framework) and with a segregated evaluation of valence, which is also hierarchical, since it relies on the same hierarchical simulation engine.

## Data Availability

No new data were created or analyzed in this study. Data sharing is not applicable to this article.

## Appendix A. Appendix: List of Abbreviations

### Appendix A.1. Brain Regions

**Acronym**	**Full Form**
ACC	Anterior Cingulate Cortex
AMY	Amygdala
BG	Basal Ganglia
dACC	Dorsal Anterior Cingulate Cortex
dmPFC	Dorsomedial Prefrontal Cortex
dlPFC	Dorsolateral Prefrontal Cortex
FEFs	Frontal Eye Fields
GP	Globus Pallidus
HC	Hippocampus
HYP	Hypothalamus
IC	Insular Cortex
IPS	Intraparietal Sulcus
MC	Motor Cortex
mPFC	Medial Prefrontal Cortex
NAc	Nucleus Accumbens
OTC	Occipitotemporal Cortex

OFC	Orbitofrontal Cortex
PVT	Paraventricular Thalamus
PAG	Periaqueductal Gray
PCC	Posterior Cingulate Cortex
PPC	Posterior Parietal Cortex
PCu	Precuneus
PFC	Prefrontal Cortex
SMA	Supplementary Motor Area
sgACC	Subgenual Anterior Cingulate Cortex
STN	Subthalamic Nucleus
TH	Thalamus
VFC	Ventral Frontal Cortex
VS	Ventral Striatum
VTA	Ventral Tegmental Area
vmPFC	Ventromedial Prefrontal Cortex

### Appendix A.2. Circuits/Pathways

**Acronym**	**Full Form**
CTC	Cerebello-Thalamo-Cortical Loop
CTSC	Cortico-Thalamo-Striatal-Cortical Loop
HPA	Hypothalamic–Pituitary–Adrenal Axis

### Appendix A.3. Functional Networks

**Acronym**	**Full Form**
AN	Attention Network
CCN	Cognitive Control Network
CEN	Central Executive Network
DMN	Default Mode Network
NAN	Negative Affect Network
PAN	Positive Affect Network
SN	Salience Network

### Appendix A.4. Other

**Acronym**	**Full Form**
AIF	Active Inference
CIT	Citalopram
CAM	Complementary and Alternative Medicine
CBT	Cognitive Behavioral Therapy
DBS	Deep Brain Stimulation
EBT	Entropic Brain Theory
ECT	Electroconvulsive Therapy
IIT	Integrated Information Theory
IPT/IPT	Interpersonal Therapy
FEP	Free Energy Principle
LTD	Long-Term Depression
LTP	Long-Term Potentiation
MOIs	Monoamine Oxidase Inhibitors
KT	Kolmogorov Theory
LSD	Lysergic Acid Diethylamide
MDD	Major Depressive Disorder
OF	Objective Function

REBUS	Relaxed Beliefs Under Psychedelics
SSRIs	Selective Serotonin Reuptake Inhibitors
SNRIs	Serotonin–Norepinephrine Reuptake Inhibitors
tDCS	Transcranial Direct Current Stimulation
tES	Transcranial Electrical Current Stimulation
TMS	Transcranial Magnetic Stimulation
TCAs	Tricyclic Antidepressants

## Appendix B. Algorithmic Specification of the Agent

Here, we provide a high-level implementation of the agent as a program.

**Algorithm A1** Algorithmic agent. The algorithm describes the interaction of the agent with the World *W* (companion agent). After initialization of Model *M* and Plan *P*, a loop is entered while valence is above zero (representing the state of being “alive”) where sensing, prediction and comparison steps update the agent’s model *M*. Valence *V* is computed from this model using the Objective Function ObjFun. The planning engine Planner takes in model and valence (of the current state), and simulates potential actions/plans *P* impacting future model and objective function values, until an optimal set of actions is found and executed.	
**Require:** *W* companion agent	
1: Model, Plan M,P= init	
2: **while** V>0 **do**	
3: I←Sense[W]	▹ Collect inputs from world
4: $I_{synth}\leftarrow \mathrm{Sim}\left[M\right]$	▹ Simulate data, make predictions
5: $e\leftarrow \mathrm{Comp}[I,I_{synth}]$	▹ Compare, compute prediction error
6: M←Updater[M,P,e]	▹ Update model
7: V←ObjFun[M]	▹ Compute valence from model
8: P←Planner[V,M;Sim,ObjFun]	▹ Formulate action plan
9: O←Act[P]	▹ Execute plan, send outputs to world
10: **end while**	

## Appendix C. Review of Brain Regions Discussed in the Text

### Appendix C.1. Nucleus Accumbens (NAc)

The nucleus accumbens (NAc), a central integrator of sensorimotor information, plays a pivotal role in facilitating either the approach or avoidance of a stimulus. It receives substantial dopaminergic input from the Ventral Tegmental Area (VTA), which releases dopamine in response to various social stimuli and motivated behaviors. The NAc also receives input from the basolateral amygdala (blAMY) and hippocampus (HIP) and projects to the ventral pallidum (VP) [302].

The NAc’s connections extend to the medial network areas, including Area 25 in the subgenual part of the medial prefrontal cortex (PFC), which specifically projects to the NAc’s “shell” [303]. The amygdala input to the striatum aligns with that of the medial network, and these striatal regions project to the ventral pallidum [303].

The NAc, along with the VP, forms part of the ventral striatopallidal system. It comprises two subregions, the core and the shell, distinguishable by hodology and neurochemical profiles. The shell contributes more to dopamine effects and behavioral reinforcement than the core. Depletion of dopamine in the NAc decreases reinforcement behaviors, while appetitive behaviors remain intact, suggesting the NAc’s role in modulating the “wanting” rather than the “liking” of relevant stimuli. Lesions to the NAc result in impulsive choices in mammals [302].

The NAc’s role in regulating behaviors related to depression and addiction underscores its potential involvement in the comorbidity of these disorders. The etiology of this comorbidity, although not fully understood, may reflect an imbalanced activation of the reward circuit and signaling mechanisms. The NAc is divided into shell and core areas, with the shell receiving limbic system information and the core primarily receiving motor system information. Its unique location and structure make the NAc critical in various motivational and emotional disorders, including depression, drug abuse, and addiction [304].

Cholinergic neurons in the NAc are crucial in the development of addiction and depression, potentially through their ability to alter CHT function, HCN2 expression, or p11 expression, and by silencing GSK3 b. Clinical studies have shown a significant reduction in p11 protein in patients with depression, suggesting p11 as a potential treatment target for comorbidity [304].

Deep brain stimulation (DBS) in the NAc has shown promise in treating addiction and depression, indicating that NAc modulation can positively impact psychiatric symptoms [304]. However, further research is needed to understand the specific mechanisms involved.

The NAc responds to the emotional intensity and self-relatedness of various stimuli, independent of their valence, with both positive and negative valences possibly processed along a rostrocaudal gradient [2]. It receives projections from midbrain regions, emotion-involved regions, motor regions, and memory-involved regions. The NAc also indirectly projects to cortical regions, including the cingular and medial prefrontal cortex, the ventral pallidum, the thalamus, the amygdala, and the hypothalamus, many of which are implicated in emotion processing [2].

The distinct roles of the NAc’s dopamine receptor D1 (D1R) and D2 (D2R) in regulating cue-elicited approach-avoidance decision-making have been highlighted in recent research. Antagonism of D1R leads to avoidance behavior, while antagonism of D2R results in preference behavior, suggesting differential control over decision-making involving approach-avoidance conflict resolution [160].

A recent study has shown that the balance of activity at NAc D1Rs plays a key role in enabling the rapid activation of a focused, reward-seeking state. This study suggests that dopamine transmission in the NAc is integral in mediating the influence of reward expectations on reward-seeking actions [305].

The NAc, in conjunction with the VTA, forms a critical part of the neurocircuitry domains that contribute to the development of compulsive-like behaviors underlying drug addiction. These domains correspond to three functional domains: binge/intoxication, withdrawal/negative affect, and preoccupation/anticipation [306], further emphasizing the NAc’s role in regulating behaviors related to addiction and depression.

### Appendix C.2. Amygdala (AMY)

The Amygdala (AMY), a significant limbic structure, plays a pivotal role in the emotional coding of environmental stimuli and in adjusting motivational levels [307]. It is particularly involved in reward processing through its interactions with the ventral striatum (VS), with connections extending throughout much of the VS and densely terminating in the nucleus accumbens (NAc) [307].

The basolateral amygdala (blAMY), a subregion of the AMY, is often referred to as the “multimodal amygdala” due to its role in integrating inputs from various sensory modalities and influencing emotional behavior. It projects to the hypothalamus, striatum, and NAc, and can modulate the firing rate of dopaminergic VTA neurons, providing a mechanistic basis for its role in modulating goal-directed behaviors. Lesions of the blAMY result in a loss of emotional learning, including fear conditioning [302].

Recent studies have shown that untreated depressed individuals exhibit amygdala hyperactivation, which has been associated with increased responses to emotional faces, particularly sad ones, in individuals with MDD [11].

The AMY is part of a system that links the medial prefrontal cortex and a few related cortical areas to the ventral striatum and pallidum, the medial thalamus, the hypothalamus, the periaqueductal gray, and other parts of the brainstem. This system is centrally involved in mood disorders, including MDD [303].

The AMY is also central to mood-congruent biases, involved in the early detection of salient information at both conscious and subconscious levels, and helps to identify the emotional significance of a stimulus and produces affective states [308]. It has numerous connections with regions important for emotion processing, including the orbitofrontal cortex (OFC) and other subcortical temporal structures, such as the hippocampus. This temporo-amygdala-orbitofrontal network modulates neural and behavioral responses to emotional stimuli, as well as integrates visceral and emotional states with cognition and behavior [308].

In MDD, the AMY shows decreased connectivity with various brain regions both within and outside of the Central Executive Network (CEN), which likely reflects a loss of top–down control [309]. This decoupling of the AMY and insula within a network involved in emotional processing has been reported in mildly depressed patients [309].

Recent insights suggest that there is a significantly reduced recurrent inhibition within the left AMY, which is consistent with the essential role of intrinsic inhibition in regulating amygdala activation and fear expression [158]. This could potentially contribute to the hyperactivity observed in the AMY in MDD patients.

Furthermore, studies have shown reduced amygdala–prefrontal coupling in major depression, which is associated with the severity of the illness [310]. This decreased functional coupling of the amygdala and supragenual cingulate is related to increased depression in unmedicated individuals with current MDD [310].

In conclusion, the AMY, including its subregion the blAMY, plays a significant role in emotional processing and reward associations in the brain. Its dysfunction could potentially contribute to the emotional dysregulation and anhedonia often observed in MDD, although further research is needed to fully elucidate these relationships.

### Appendix C.3. Dorsolateral Prefrontal Cortex (DLPFC)

The DLPFC is a pivotal brain region with distinct functional roles. It can be divided into three regions—dorsal, ventral, and caudal—based on local cortico-cortical connections, each interconnected with other local areas and connected to specific areas in other parts of the cortex [303]. Further subdivision, as per the Sallet atlas, reveals ten subregions grouped into three main areas: a frontal part, a medial part, and a posterior part [311,312].

In Major Depressive Disorder (MDD), the DLPFC plays a significant role, with dysregulation potentially disrupting goal-directed cognition and working memory [193]. The DLPFC is involved in cognitive control, working memory, and selective attention, with hypoactivation often leading to cognitive deficits in MDD. However, hyperactivation, potentially a compensatory mechanism, and structural alterations such as reduced gray matter have also been observed [11].

The DLPFC exhibits a stable and individual-specific functional connectivity pattern, which remains consistent within individual subjects across different days, suggesting its potential as a biomarker in MDD diagnosis and treatment [184]. As a key component of the Central Executive Network (CEN), the DLPFC plays a crucial role in working memory and attention, co-activating with the Salience Network (SN) during various cognitive tasks [56].

The DLPFC is also a key component of the Cognitive Control Network (CCN), which is prominently disrupted in late-life depression (LLD), resulting in symptoms of executive dysfunction [313]. Furthermore, the DLPFC is part of the left and right DLPFC/parietal cortex networks, which impose strong constraints on information processing [56].

The DLPFC is also a node within the Central Executive Network (CEN), which has been shown to be dysregulated in patients with depression [314]. A study by Camchong et al. suggested that tDCS targeting the DLPFC could potentially be used as a therapeutic intervention for alcohol use disorder (AUD), and by extension, for MDD [315].

The DLPFC has been identified as a potential target for individualized transcranial magnetic stimulation (TMS) treatment [184] and is involved in modulating thalamocortical and cortico-thalamic activity, which could be related to the difficulty MDD patients have in “letting go” of a negative mood [303]. It also plays a role in reward processing, particularly in monitoring incentive-based behavioral responses, and in the control of goal-directed and stimulus-driven attention [157,307].

The DLPFC’s functional connectivity is modulated by emotional states, potentially contributing to mood dysregulation in MDD [316]. Altered functioning in the DLPFC, indicative of aberrant working memory processing, has been observed in MDD, highlighting its potential as a therapeutic target [310]. The DLPFC is also thought to regulate memory-relevant cortical activity levels, influencing memory representation [317]. The DLPFC has been implicated in the consolidation and transformation of episodic memory, with longitudinal studies showing increased connectivity between the DLPFC and the hippocampus predictive of memory retention [317].

In conclusion, the DLPFC is a complex and multifaceted region of the brain, with a significant role in cognitive function and the pathophysiology of MDD. Understanding the specific roles of its subregions and its individual-specific functional connectivity could provide valuable insights into the etiology of MDD and inform the development of novel therapeutic approaches.

### Appendix C.4. Hippocampus (HC)

The hippocampus, a key component of the human brain, is instrumental in the formation of new, declarative memories, encompassing both episodic and semantic memories. Episodic memory pertains to the recall of specific events linked to a particular time and place, while semantic memory relates to general knowledge about the world. The hippocampus is deeply involved in these memory processes [121].

Moreover, the hippocampus facilitates communication between the neocortex and itself, processing “what” (e.g., stimuli, items) and “where” information in segregated parallel streams. This functional segregation is preserved in the parahippocampal region, with the perirhinal and lateral entorhinal areas crucial for item memory (“what”), and the postrhinal and medial entorhinal areas significant for contextual memory (“where”) [133]. The hippocampus also plays a pivotal role in integrating event and context information, forming specific item–place associations and integrating what–where information. This ability is reflected in the coding properties of individual hippocampal neurons, with different subsets selectively coding for “what” and “where” information and others for specific what–where conjunctions [133]. The hippocampus is also implicated in forming what–when associations, including memory for the sequence of specific events, a capacity primarily dependent on its subregion CA1 [133].

Beyond memory formation, the hippocampus is intricately connected to various other cortical and subcortical regions of the brain, receiving inputs from and sending outputs to several areas [121]. This suggests that the hippocampus is part of a complex brain network, contributing to its diverse functions.

The hippocampus selectively contributes to the retrieval of detailed context memories [103] and is involved in the consolidation of information during memory consolidation, forming a cortical–hippocampal–cortical loop of information processing [103].

The role of the hippocampus in depression is not explicitly mentioned in the provided snippets, necessitating further research. Similarly, information on specific symptoms or patient subtypes associated with hippocampal dysfunction is not provided. However, a study by Dedovic et al. (2010) found a significant positive correlation between hippocampal volume and cortisol awakening response (CAR) in men, suggesting a potential role of the hippocampus in stress response, often dysregulated in depression [318].

### Appendix C.5. Orbitofrontal Cortex (OFC)

The orbitofrontal cortex (OFC) is a pivotal brain region involved in a myriad of functions, including sensory processing, pleasure coding, and executive function. Functionally, it is part of two distinct networks: the sensory-related orbital network and the output-oriented medial network, which modulates visceral function in relation to emotion and other factors [303].

The OFC is also a crucial hub for pleasure coding, with its subregions playing distinct roles in hedonic processing [3]. Neuroimaging studies have revealed differential activity in the medial OFC (mOFC), mid-anterior OFC (midOFC), and lateral OFC (lOFC) in response to reward, depending on the context [3].

Moreover, the OFC, in conjunction with the ventromedial prefrontal cortex (vmPFC) and the dorsal anterior cingulate cortex (dACC), projects to specific regions within the ventral striatum (VS), providing an anatomical substrate for modulation between these circuits [307]. This suggests a critical role for the OFC in integrating information from different cortical areas before passing it onto the medium spiny projection cells [307].

The OFC’s role extends to sensory processing, with parts of the OFC, such as the lateral OFC, projecting directly to all sensory cortices, thereby directly influencing sensory processing [319]. This suggests that the OFC is involved in the integration of sensory information and affective states, which could be disrupted in Major Depressive Disorder (MDD) [310].

In the context of MDD, the OFC, along with the medial prefrontal cortex (mPFC), has been found to show abnormalities that emerge with the onset of MDD. Structural MRI studies have reported volumetric reductions in the OFC in patients with MDD, indicating a potential link between MDD and gray matter volume reduction in the OFC [310].

The OFC is also implicated in executive function networks related to addiction, specifically in the Executive Function Stop (EF-Stop) network [320]. This suggests that the OFC may play a role in inhibitory control, which could be relevant in the context of MDD, given the high comorbidity between depression and addiction disorders.

Further studies have shown that depressed patients exhibit increased OFC functional connectivity with the dorsolateral prefrontal cortex (DLPFC) in response to negative stimuli, which may reflect a greater neural response to such stimuli [308]. Additionally, reduced OFC connectivity with the precuneus and cingulate gyrus has been observed, which may be associated with problems in regulating self-schemas [308].

The OFC is also implicated in compulsivity, a feature of several disorders, including Obsessive Compulsive Disorder (OCD), substance dependence, eating disorders, and behavioral addictions [49]. In OCD patients, brain imaging shows abnormal activity in the OFC and other regions, which are known to be important for assessing whether stimuli are positive or negative (rewarding or punishing) and for habit-learning [49].

The OFC also mediates outcome encoding in Pavlovian conditioning, which is a learning process that could be disrupted in MDD. Moreover, aberrant working memory processing, which is associated with the OFC, has been observed in major depression [310].

The OFC is also involved in attention and cognitive control circuits, which are implicated in higher cognitive functions such as working memory and selective attention [10]. Dysfunction of these circuits during the effortful selective processing of relevant stimuli while inhibiting irrelevant stimuli suggests a cognitive dyscontrol biotype in depression, which could be associated with OFC dysfunction [10].

In conclusion, the OFC is a critical region of the brain that plays a significant role in emotion, visceral function, pleasure coding, executive function, compulsivity, and cognitive control.

### Appendix C.6. Insular Cortex (IC), Insula

The insular cortex, or insula, is a significant component of the brain’s functional network, playing a pivotal role in interoception, a sensory-driven phenomenon associated with the neuroanatomy of ascending homeostatic pathways that transmit interoceptive sensations to the brain [157]. The insula is instrumental in converting these signals into a ‘global emotional moment’, with the anterior insula being a central area for human awareness [157].

The insula is also a part of the mesocorticolimbic circuit, a key player in processing diverse pleasures. Neuroimaging studies frequently highlight the insula’s activation in response to various pleasures, indicating its role in generating hedonic experiences, which are fundamental to emotional well-being [321].

Furthermore, the insula is integrated into the medial network, which is interconnected with other areas such as the anterior and Posterior Cingulate Cortex, and the entorhinal and parahippocampal cortex. This network is characterized by outputs to visceral control areas in the hypothalamus and periaqueductal gray, modulating visceral function in relation to emotion or other factors [303].

The insula is also recognized as one of the “rich club” hubs, believed to function as a high-capacity backbone for synchronizing neural activity and integrating information across the entire brain [52].

In the context of Major Depressive Disorder (MDD), the insula’s role remains to be fully elucidated. However, its involvement in emotional processing and awareness, as well as in the generation of hedonic experiences, suggests a potential role in the etiology of MDD. Insular dysfunctions could potentially lead to altered emotional awareness and processing and a diminished capacity for pleasure, common symptoms in MDD patients [10].

The insula’s role in dynamically mapping changes in cardiorespiratory interoception could also be relevant in understanding its role in MDD [262]. Symptoms such as anhedonia, persistent feelings of sadness, or emotional numbness, often seen in MDD patients, could potentially be linked to insular dysfunction [10].

Interestingly, emotional awareness can be preserved even after extensive bilateral damage to the insula, anterior cingulate, and amygdala [157], suggesting a more complex role of the insula in emotional processing and awareness than previously thought.

The insula is also part of the intrinsic cognitive networks (ICNs) of the human brain, which impose strong constraints on information processing. Disruptions to these networks contribute to specific patterns of cognitive and behavioral impairments [56], further emphasizing the potential role of the insula in the etiology of MDD and its potential as a target for therapeutic interventions.

In late-life depression, cerebrovascular disease may predispose, precipitate, or perpetuate a depressive syndrome. The presence of white matter lesions identified as white matter hyperintensities (WMH) on T2-weighted or fluid-attenuated inversion recovery MRI is associated with a worse illness course and cognitive performance. Moreover, greater WMH volume was associated with increased BOLD response in the rostral cingulate (including the sgACC) and insula in older adults with major depression when completing an emotional regulation task [313]. In conclusion, the insula’s crucial role in the brain’s functional network, particularly in emotional processing and awareness, and in the generation of hedonic experiences, suggests its potential involvement in the etiology of MDD and its potential as a target for therapeutic interventions.

### Appendix C.7. Ventromedial Prefrontal Cortex (vmPFC)

The vmPFC is a critical node in the brain’s functional neuroanatomy, playing a pivotal role in fear regulation and extinction, with its neurons signaling memory for fear extinction. It is a critical component of the brain’s neural circuitry, playing a significant role in the regulation of emotional responses and stress [303]. It is part of the medial prefrontal network, which modulates visceral responses via the HYP and brainstem, and is considered part of a larger “default system” of cortical areas implicated in self-referential functions [303]. This network, including the vmPFC, is interconnected and characterized by outputs to visceral control areas in the HYP and periaqueductal gray, forming an output system that modulates visceral function in relation to emotion or other factors [303]. It is also part of the core neurocognitive networks, including the Central Executive Network (CEN), Salience Network (SN), and Default Mode Network (DMN), playing a crucial role in self-referential mental activity and the detection and mapping of salient external inputs and internal brain events [56]. Moreover, the ventromedial PFC is implicated in autobiographical memory retrieval, with strong connectivity with the hippocampus, crucial for memory function, particularly pronounced during the early construction phase of detailed remote memories [317].

The vmPFC plays a role in reward-based learning, receiving inputs from the DLPFC and projecting to NAcc, a region involved in reward and reinforcement learning [307]. This connection suggests a role for the vmPFC in the regulation of reward and motivation, which may be disrupted in MDD [307]. The vmPFC is implicated in the generation of an abstract representation of the rewarding value of a stimulus by attending to its context, and the learning of contingencies based on this representation [2]. This function may be particularly relevant to the anhedonia often observed in MDD. The vmPFC supports the ability to focus on goal-relevant information by filtering out irrelevant information, a process akin to dimensionality reduction [322]. It is also involved in autobiographical memory retrieval, showing strong connectivity with the hippocampus during the retrieval of detailed remote memories [317].

The vmPFC, specifically the medial prefrontal cortex (mPFC) region, responds to rewarding outcomes and to contextual aspects of reward during anticipation [307]. vmPFC has been identified as a connector hub of the dorsal subnetwork, interacting with the medial fronto-polar cortex (mFP) via sgACC in the ventral subnetwork [316]. It is also part of the Default Mode Network (DMN), involved in various cognitive processes such as semantic and conceptual processing, imagery, mind wandering, memory, empathy, person perception, mental-state inference, self-related processing, valuation, and language processing [157]. As part of DMN, the vmPFC has strong structural connections to limbic areas such as AMY, and has been linked to emotional control, self-referential activity, and internal mentation [309]. The DMN is crucial for self-referential mental activity, and vmPFC dysfunction could contribute to the self-focused negative rumination often seen in MDD [56].

The ventromedial PFC’s role extends to interoceptive predictions, fundamental to perception, cognition, and emotion. Aberrant interoceptive predictions can lead to chronic physical burdens, resulting in disorders of mood, appetite, and metabolism, such as depression [157]. This region also acts as a connector hub in the brain’s dorsal subnetwork, interacting with the medial fronto-polar cortex (mFP) via the sgACC in the ventral subnetwork, with these connectivity patterns associated with the intensity of sadness experienced by individuals [316].

This region is also implicated in the etiology of MDD, with distinct roles in depression attributed to the vmPFC and the DLPFC [101,185]. The vmPFC is associated with self-referential thinking and emotion regulation: hyperactivity in the vmPFC has been linked to rumination, a common symptom of depression where individuals persistently focus on their depressive symptoms or the causes of their distress. This would commonly be associated with biotypes of depression characterized by high levels of rumination or negative self-focus. The DLPFC, on the other hand, is involved in cognitive control and executive function. Hypoactivity in the DLPFC is often associated with depression, contributing to symptoms such as difficulty concentrating or making decisions. This could align with biotypes of depression that display pronounced cognitive symptoms.

The vmPFC is implicated in the pathophysiology of depression, with structural abnormalities and chronically hyperactive metabolism within the vmPFC and other agranular visceromotor regions preceding the onset of depression [157]. In MDD, the vmPFC and related regions exhibit alterations in gray matter volume, cellular elements, neurophysiological activity, receptor pharmacology, and gene expression [303]. Dysfunction within this network is posited to underlie the disturbances in emotional behavior and other cognitive aspects of MDD, including disturbances in autonomic and neuroendocrine function [303]. The ventromedial PFC’s dysfunction is particularly relevant in depression subtypes associated with chronic alcohol use, which can remodel prefrontal neurons and disrupt NMDA receptor-mediated fear extinction encoding [185]. This region is also associated with reward processing, often dysfunctional in MDD, and linked to anhedonia, a core symptom of depression [323].

The ventromedial PFC’s association with autonomic function suggests that its dysfunction may contribute to the heightened affective, neuroendocrine, and sympathetic autonomic arousal seen in depression, potentially leading to an understimulation of parasympathetic tone in mood disorders [324].

### Appendix C.8. Anterior Cingulate Cortex (ACC)

The anterior cingulate cortex (ACC), particularly the subgenual ACC (sgACC), plays a pivotal role in emotional behavior modulation, as evidenced by neuroimaging studies in humans and lesion analyses in experimental animals [324]. This region, also known as the subcallosal anterior cingulate gyrus, is composed of the agranular cortex, characterized as the Brodmann area (BA) 24 anteriorly and BA 25 posteriorly [324].

In Major Depressive Disorder (MDD), the sgACC exhibits abnormal reductions in gray matter volume, irrespective of mood state, in subjects with MDD and bipolar disorder. This abnormality is associated with a reduction in glia, without a corresponding loss of neurons. Moreover, metabolic activity in the sgACC is elevated during depressive phases in MDD subjects, with effective antidepressant treatment correlating with a reduction in sgACC activity [324].

The sgACC’s clinical significance in MDD is underscored by its role in ameliorating depressive symptoms in treatment-resistant MDD through deep brain stimulation [324]. Furthermore, the ACC is a critical hub for pain-induced depression, emphasizing its role in emotional regulation and depressive symptom manifestation [325].

The ACC, receiving nociceptive information from thalamic inputs, mediates cognitive and affective aspects of pain [325]. Overactivation of the sgACC enhances cardiovascular, behavioral, and neural responses to threat, altering cortisol dynamics during stress, reducing vagal tone and heart rate variability, and heightening reactivity to proximal and distal threat [185].

The ACC, including the sgACC, is part of the negative affect circuit, which is engaged by negative emotional stimuli and modulated by serotonergic neurotransmitter pathways [326]. The sgACC is implicated in sadness regulation, possibly through its regulatory role in relation to two hub network areas, rather than a direct driving mechanism [316].

The sgACC has been found to be a useful predictor of antidepressant treatment outcome, providing insight into which patients with depression might respond to treatment [314]. The ACC, including the sgACC, is connected to several brain structures via white matter tracts such as the cingulum bundle and uncinate fasciculus, which have been strongly implicated in the Salience Network (SN) and serve as potential sites of modulation for emotion dysregulation [314].

In conclusion, the sgACC’s crucial role in emotional behavior regulation and its significant impact in MDD, particularly in treatment-resistant MDD and pain-induced depression, underscores its importance. Overactivation of the sgACC can lead to enhanced responses to threat, further emphasizing its role in stress-related disorders such as depression and anxiety.

### Appendix C.9. Ventral Tegmental Area (VTA)

The Ventral Tegmental Area (VTA), a critical component of the brain’s reward circuitry, is primarily composed of dopamine neurons and is centrally located within the brain’s reward circuit, which also includes the nucleus accumbens (NAc) and the substantia nigra (SN) among other regions [307]. The VTA is part of the mesolimbic dopamine system, which includes dopamine cell bodies within the midbrain VTA and substantia nigra (SN) that send reciprocal projections throughout the limbic brain, including the striatum, amygdala, habenula, hippocampus, and prefrontal cortex (PFC) [325]. This system is crucial for reward processing, but it does not drive the hedonic experience of reward (“liking”) but rather the instrumental behavior of reward-driven actions (“wanting”) [325].

The NAc, which receives complex inputs and outputs from multiple brain regions including the VTA, plays a critical role in a variety of motivational and emotional disorders, including depression, drug abuse, and addiction [163]. The main output neurons from the NAc are medium spiny neurons (MSNs), which project via GABAergic projections to the VTA, among other regions [163]. MSNs are divided into two types according to dopamine (DA) receptor expression: the DA receptor D1 positive (D1C) MSNs stimulate reward-related behavior while DA receptor D2 positive (D2C) MSNs promote aversive behavior [163].

The VTA is also a part of the social decision-making network (SDM), which is concerned with regulating and implementing adaptive behavioral outputs in response to salient environmental challenges and opportunities. This network shares overlapping nodes with the mesolimbic reward system and the social behavior network (SBN), and has been studied extensively in the context of addiction and regulating appetitive behavior [302].

While the specific role of the VTA in depression is not explicitly mentioned in the provided snippets, it is well established that dysfunctions in the brain’s reward circuitry, including the VTA, are implicated in Major Depressive Disorder (MDD). Alterations in the reward circuit can lead to anhedonia, a core symptom of MDD characterized by a diminished ability to experience pleasure. Reduced phasic dopamine signaling, a hallmark of anhedonia, is associated with the VTA and is exhibited in both drug-dependent and chronic pain states [325].

Clinical studies of individuals with mood disorders have implicated that the networks are formed by the orbitomedial prefrontal cortex (OMPFC) and anatomically related areas of the striatum, thalamus, temporal cortex, and limbic system in the pathophysiology of depression. These networks, which include the VTA, contain alterations in gray matter volume, cellular elements, neurophysiological activity, receptor pharmacology, and gene expression in individuals with mood disorders. Dysfunction within these circuits may account for the disturbances in emotional behavior and other cognitive aspects of the major depressive syndrome [303].

Xu et al. provide additional insight into the role of the VTA in depression, particularly in relation to addiction. It suggests that the VTA Cav1.3 channels are necessary for a GluA1 S831 phosphorylation-dependent increase in CP-AMPARs in the NAc shell, a mechanism that has been suggested to contribute to increases in CP-AMPAR levels at the synapse. This Cav1.3/CaMKII/ERK2 signaling within the VTA mediates the long-term molecular changes in the NAc via a CREB-dependent mechanism. Moreover, cocaine CPP and depressive-like phenotypes can be reversed by the infusion of CP-AMPAR-specific blockers into the NAc shell, which suggests that the modulation of AMPAR transmission may link addiction with depression [163].

Gorwood et al. [2] further emphasize the role of the VTA in anhedonia, a core symptom of MDD. It suggests that the ventromedial prefrontal cortex (VMPFC), which is involved in the cognitive aspects of emotional processing, may reflect a cortical compensatory mechanism for an underactive subcortical/striatal response to pleasant stimuli. This could be due to the VMPFC inhibiting emotional processing performed by other limbic structures, leading to reduced emotional experience. Furthermore, the snippet suggests that the VTA and other regions involved in the identification of emotional stimuli and the generation of emotional behavior show increased activity in depressed patients, while regions involved in the effortful regulation of emotional behavior show decreased activity. This pattern of activity reverses after recovery from a major depressive episode.

Price et al. [303] provide additional information on the connections of the VTA with other brain regions. It suggests that the VTA is connected to the medial prefrontal network, which has substantial outputs to the hypothalamus, the periaqueductal gray, and other visceral control centers. These connections may play a role in the regulation of emotional responses and the assessment of non-food sensory objects.

Di Giovanni et al. [327] suggest that serotonin-2C receptors play a role in the function of the VTA. This could potentially link the VTA to the serotonin system, which is known to be involved in mood regulation and is often targeted in the treatment of depression.

### Appendix C.10. Hypothalamus (HYP)

HYP, a small but crucial region at the brain’s base, is integral to autonomic functions such as heart rate and respiration. It is located above the brainstem and below TH. It is roughly the size of an almond. This region plays a crucial role in many important functions, including releasing hormones and regulating body temperature, hunger, thirst, sleep, and circadian rhythms. It has direct connections with various other brain regions that are integral to emotional processing, including AMY, which processes fear and anxiety, and HC, involved in memory formation.

HYP plays an important role in the complex neural circuits responsible for the processing and evaluation of valence—the intrinsic attractiveness (positive valence) or averseness (negative valence) of an event, object, or situation.

It receives and integrates diverse signals from various brain regions and subsequently helps evaluate the emotional or hedonic value (valence) of stimuli. These evaluations can then inform motivated behaviors. Signals from HYP are further processed by regions such as the NAc, a key component of the brain’s reward system, to generate the overall perception of reward.

At the same time, HYP also plays a critical role in encoding actions related to the reward system. Specifically, the lateral HYP has been found to be particularly involved in reward-seeking behaviors. As we saw, these behaviors can be broadly classified into two types: “liking” or “wanting”. “Liking” behaviors refer to the immediate pleasure derived from a reward, while “wanting” behaviors refer to the motivational component driving an organism to seek out a reward. HYP is thought to be particularly involved in the “wanting” aspect of reward processing (action).

Motivations or emotions generated by subcortical circuits involving the NAc may be powerfully modulated by “top-down” corticolimbic controls from PFC. In humans, the successful voluntary suppression of subjective cravings or emotional responses is accompanied by the activation of prefrontal cortical areas and a simultaneous reduction in subcortical activity in NAc, AMY, and VTA. In this context, the HYP modulates and complements inputs from PFC to NAc, and as such, it may be seen to be part of the agent’s distributed valence evaluation system [155].

HYP plays a key role in reward processing. The hypothalamic pathway, particularly the lateral hypothalamic area, is intimately connected with the reward centers in the brain—both evaluation and action. Lateral HYP to VTA neurons encode the learned action of seeking a reward, independent of reward availability, driving action [328]. These neurons selectively encoded the action of reward-seeking, but did not encode environmental stimuli, while rewarding stimuli and reward-predictive cues were encoded by a discrete population of lateral HYP neurons downstream of the VTA [328].

This area is associated with pleasure and reward behaviors. The hypothalamus is part of the larger mesolimbic dopamine system, commonly referred to as the brain’s “reward pathway”. It belongs to a network of brain regions, most notably involving VTA and NAc, mediating reward seeking and consumption behavior by increasing the saliency of environment cues and promoting positive reinforcement [328]. HYP is heavily influenced by the medial prefrontal network, particularly the subgenual cortex, with projections terminating in both the medial and lateral hypothalamus and the periaqueductal gray (PAG), a region implicated in pain modulation and defensive behavior [303].

Lesions in the ventromedial prefrontal cortex (vmPFC), which is strongly connected to the HYP, can eliminate the automatic visceral response to emotional stimuli. This can lead to severe decision-making impairments, despite preserved cognitive intelligence, resulting in a failure to comprehend the long-term implications of actions and a propensity for immediate gratification [303]. HYP is also part of a complex network with the TH and PFC, involved in reward processing. Dysfunction within this network could contribute to depressive symptoms. Evidence from PET and fMRI studies suggests that HYP may be implicated in reward-processing deficits observed in depression [307].

Intersecting with the social behavior network (SBN) and reward systems, the hypothalamus plays a fundamental role in behavior regulation and is part of a conserved social decision-making circuit across major vertebrate lineages, integrating social and reward information [302]. The lateral septum (LS) and bed nucleus of the stria terminalis (BNST), both hypothalamic components, mediate information about social stimulus salience into adaptive behavioral output, regulating social and reward-related behaviors. HYP also regulates parental care and sexual behavior, with lesions inhibiting receptivity and preventing parturition in certain animals, suggesting a role in reproductive behaviors [302].

HYP is implicated due to its role in stress response. Dysregulation of the Hypothalamic–Pituitary–Adrenal (HPA) axis, which controls the body’s response to stress, is a key factor in the pathophysiology of depression. Chronic stress can lead to hyperactivity of the HPA axis, which can result in depressive symptoms. Furthermore, the hypothalamus’ role in sleep and circadian rhythm regulation can also contribute to mood disorders, as disruptions in these areas are commonly seen in depression.

Interactions between HYP and mesocorticolimbic reward systems have been highlighted in recent research. For instance, hypothalamic orexin–hypocretin neurons modulate the NAc, potentially enhancing food reward during hunger states. These interactions could play a significant role in modulating food reward during hunger and satiety states and may contribute to the development of eating disorders. Moreover, lesions of the lateral HYP disrupt eating and drinking behaviors, suggesting a crucial role in regulating food “wanting” and “liking”. However, the complexity of the hypothalamus and its interconnectedness with other brain regions, such as the ventral pallidum, have been underscored by these findings [329].

Furthermore, the hypothalamus, along with other visceromotor regions, is involved in allostasis, the regulation of the body’s resources. Prolonged imbalances can lead to illnesses that remodel the brain and the sympathetic nervous system, leading to behavioral changes. This could potentially explain the role of the hypothalamus in depression [52]. In conclusion, dysfunction in the hypothalamus or its connecting circuits to the PFC could contribute to depressive symptoms.

### Appendix C.11. Thalamus (TH)

The thalamus, a critical “relay station” in the brain, is instrumental in transmitting sensory and motor signals to the cerebral cortex, regulating states of sleep and wakefulness, and modulating consciousness and alertness. Its role extends to the pathophysiology of Major Depressive Disorder (MDD), where it is implicated in mood and emotion dysregulation, key features of the disorder [157].

The thalamus’s central role in thalamocortical oscillations is particularly noteworthy. Increased thalamic excitation can disrupt these rhythms through its extensive projection to the neocortex, a phenomenon observed in MDD patients. This disruption can lead to symptoms such as insomnia, anhedonia, and cognitive impairment, all associated with MDD [158]. The thalamus modulates the corticothalamic network, influencing states of consciousness, vigilance, and higher cognitive processes. This modulation is mediated by serotonin (5-HT) via its receptors, which are widely expressed in the corticothalamic network and areas involved in slow-wave-discharge modulation, such as the striatum, nucleus accumbens, and the substantia nigra pars reticulata [327].

The thalamus’s connections extend to the midline nuclei, including the paraventricular thalamic nucleus, and other nuclei situated between the anterior and mediodorsal thalamic nuclei. These nuclei have extensive connections, particularly with the medial prefrontal network areas, and relay visceral function information to both the medial prefrontal network and the cortico-striato-pallido-thalamic loop [303].

The thalamus’s significant connection with the prefrontal cortex (PFC), another region implicated in MDD, is also noteworthy. The mediodorsal nucleus of the thalamus projects to the orbital and medial prefrontal cortex, suggesting a potential pathway for thalamic influence on mood and cognitive functions [307].

The thalamic relay nuclei from the basal ganglia integrate information flow from reward and higher cortical “association” areas of the prefrontal cortex, which could be disrupted in MDD, leading to attentional deficits [307]. Both PET and FMRI findings suggest that primary and secondary rewards can increase thalamic activation, indicating a potential role of the thalamus in reward processing, often impaired in MDD [307].

The thalamus’s strong connections with the amygdala, a structure involved in emotion generation and experience, further underscore its role in emotional processing [303]. In terms of neural oscillations, the thalamus may influence the formation of semantic representations through cortico-cortical interregional interactions, which could be disrupted in MDD, affecting the integration of new information into existing knowledge [317].

### Appendix C.12. Caudate (CAU)

The medial caudate, a key component of the brain’s reward system, is instrumental in strategic action planning. It synthesizes information from reward and cognitive cortical areas, thereby facilitating the selection of the most advantageous subsequent action. This function is particularly pronounced when reward feedback informs subsequent actions, but diminishes as action requirements become more predictable [307].

The caudate is embedded within the striato-nigro-striatal network, a sophisticated system of connections encompassing various functional regions of the striatum, including the ventral striatum (VS), the dorsal striatum, and the midbrain dopamine cells. This network is organized in an ascending spiral, facilitating a feed-forward organization from reward-related regions of the striatum to cognitive and motor areas [307].

In relation to Major Depressive Disorder (MDD), research has identified smaller putamen and caudate volumes in MDD patients compared to controls, indicating potential structural abnormalities in these regions [303]. However, this finding is not universal, with some studies reporting no significant difference in striatal or pallidal volumes between younger MDD subjects and controls [303].

Given the caudate’s role in reward processing and its interconnectedness with other functional regions of the striatum, it is conceivable that dysfunction in this area could contribute to the anhedonia (loss of interest or pleasure in activities) frequently observed in MDD patients. Furthermore, the caudate’s role in action control, as evidenced by rodent studies, may also be relevant to its function in humans, potentially linking caudate dysfunction to symptoms of impulsivity and poor decision-making in MDD patients [330].

While the specific symptoms and patient subtypes associated with caudate dysfunction are not detailed in the provided snippets, it is reasonable to infer that individuals with MDD who exhibit pronounced anhedonia or impulsivity may have abnormalities in caudate function.

### Appendix C.13. Pallidum

The pallidum, a central brain region, is implicated in mood disorders due to its connections with the medial network areas, including the nucleus accumbens and the medial edge of the caudate nucleus, which are particularly significant in Major Depressive Disorder (MDD) [303]. The pallidum’s role in emotional dysregulation, a characteristic of MDD, is suggested by its receipt of projections from the striatum, which is in turn influenced by the amygdala, a region associated with emotional responses [303].

The pallidum’s involvement in the cortico-striato-pallido-thalamic loop, which interacts with the medial prefrontal network and relays visceral function information, could potentially be disrupted in MDD, leading to symptoms such as anhedonia and appetite changes [303].

The ventral pallidum (VP), a pallidum component, mediates the hedonic impact of ”liked” sensations, suggesting a role in pleasure experience. Activation of the VP’s opioid system can increase the hedonic impact of natural taste rewards and cause rats to eat over twice as much food. Conversely, activation of the VP’s anterior portion, whereby disgustingly rotten food sight has been reported to activate in humans, can suppress “liking” reactions and reduce eating behavior [329].

The VP’s involvement in the motor output of motivated or goal-directed behaviors and its role in reward processing, mediating both the “liking” and “wanting” components of reward, suggest that dysfunction in this area could contribute to the anhedonia and motivation changes observed in MDD [302].

The basolateral amygdala (blAMY), which projects to the pallidum, is involved in emotional behavior and modulates goal-directed behaviors. Lesions of the blAMY result in a loss of emotional learning, including fear conditioning [302], suggesting that dysfunction in the pallidum-blAMY circuit could contribute to the emotional dysregulation observed in MDD.

The pallidum’s serotonin (5-HT) receptors play a role in distinct behavioral responses, with their expression dependent on the network’s activity state. Stimulation of 5-HT2C receptors can dampen impulsive responses and promote compulsive responses, and both agonists and antagonists at 5-HT2C receptors have been proposed in the treatment of mood disorders, including MDD [327].

However, the specific symptoms and patient subtypes associated with pallidum dysfunction in MDD are not clearly defined and may require further research.

### Appendix C.14. Putamen

The putamen, a central brain structure within the striatum, plays a pivotal role in various brain circuits and networks, including the regulation of movements, learning, and the brain’s reward system [303]. It receives inputs from numerous brain areas, such as the lateral prefrontal cortex (LPFC), which projects to the striatum’s central and dorsal parts [303].

In the context of Major Depressive Disorder (MDD), the putamen’s role is less explicit. However, it is implicated in mood disorders due to projections from the medial network areas to the nucleus accumbens and the adjacent medial edge of the caudate nucleus bordering the lateral ventricle, which encompasses parts of the putamen [303]. This suggests a potential role of the putamen in depressive symptomatology, warranting further investigation.

Research has indicated smaller putamen and caudate volumes in MDD patients compared to controls, implying a structural abnormality in these areas in depression [303]. However, this finding is not universal across all patient subtypes or age groups, with some studies reporting no significant difference in striatal or pallidal volumes between younger MDD subjects and controls [303].

The putamen’s role in the brain’s reward system is particularly significant, as it forms part of a complex network mediating various aspects of incentive-based learning, leading to adaptive behaviors [307]. This network interfaces with circuits mediating cognitive function to influence motor planning, suggesting that putamen dysfunction could disrupt this process and contribute to MDD symptoms [307].

The putamen is also part of the salience network, crucial for detecting and mapping salient external inputs and internal brain events [56]. This network’s extensive connectivity with subcortical and limbic structures involved in reward and motivation underscores the putamen’s role in the brain’s reward system, potentially explaining its involvement in MDD, where reward processing dysfunctions are a key feature.

Anhedonia, a common symptom in depression characterized by a reduced ability to experience pleasure, is thought to be related to abnormalities in reward processing. fMRI studies have found that depression is characterized by reduced responsivity in the brain’s cortical–basal ganglia reward network, which includes the putamen [308].

The specific symptoms and patient subtypes associated with putamen dysfunction in MDD are not clearly defined. However, given its involvement in the reward system and movement regulation, it is plausible that putamen dysfunction could contribute to symptoms such as anhedonia and psychomotor retardation, both common in MDD.

### Appendix C.15. Globus Pallidus (GP)

The Globus Pallidus (GP), a subcortical structure within the brain, is integral to the regulation of voluntary movement as part of the basal ganglia. This group of nuclei is associated with a myriad of functions, including control of voluntary motor movements, procedural learning, habit learning, eye movements, cognition, and emotion [307]. The GP is also part of the reward circuit, a complex network of cortical and subcortical regions that mediate incentive-based learning and adaptive behaviors. This circuit interfaces with cognitive function circuits to influence motor planning, demonstrating the existence of dual cortico-basal ganglia systems that allow for both parallel and integrative processing. These interactive networks channel reward information through cognitive circuits to influence motor control [307].

The GP primarily receives projections from the VS, a region linked with reward and motivation. These fibers terminate in specific areas of the GP, namely the subcommissural ventral pallidum, the rostral pole of the external segment, and the rostromedial portion of the internal segment. However, the central and caudal portions of the GP are devoid of this input [307].

It is suggested that increased GP inputs could disrupt ongoing patterns, facilitating a behavioral switch. This could encompass behaviors related to mood, object value assessment, and stimulus–reward association. Notably, lesions in the ventral striatum and pallidum have been linked to perseverative deficits in stimulus–reward reversal tasks in rats and monkeys, which could parallel the difficulty in mood disorders to “let go” of a negative mindset [303]. Aberrant connectivity in the GP has been associated to a common core of connectivity alterations in MDD [101].

The ventral pallidum, a component of the GP, has been implicated in mediating the hedonic impact of “liked” sensations. Activation of the opioid system in the posterior ventral pallidum can enhance the hedonic impact of natural taste rewards and increase food consumption in rats. Conversely, activation in the anterior portion can suppress “liking” reactions and reduce eating behavior, suggesting a potential role of the GP in mediating pleasure and reward responses [329].

### Appendix C.16. Periaqueductal Gray (PAG)

The Periaqueductal Gray (PAG) is a pivotal component of the brain’s circuitry, instrumental in the regulation of visceral functions [303]. It receives substantial outputs from the medial prefrontal network, including the sgACC and the lateral part of the medial network. The subgenual cortex provides the most substantial projection, terminating in both the medial and lateral hypothalamus, and in both dorsolateral and ventrolateral columns of the PAG. The fibers from the lateral part of the medial network are restricted to the lateral hypothalamus and the ventrolateral PAG [303].

The medial prefrontal network, which heavily projects to the PAG, has been associated with visceral activation in response to emotional or non-emotional stimuli. This suggests that the PAG, as a recipient of these projections, may play a role in the emotional dysregulation observed in MDD [303].

PAG dysfunction could potentially contribute to symptoms associated with decision-making, favoring immediate reward without considering long-term consequences. This is due to the strong connection between the PAG and the vmPFC, where lesions can abolish the normal, automatic visceral response to emotive stimuli [303].

The PAG also modulates certain behaviors in animals, such as nursing and aggression in female rats, and plays a distinctive role in vocalizations, specifically call initiation. It is active during human speech, and lesions of the PAG can lead to mutism [302].

The GABAergic system, rich in neuronal projections in several brain regions, including the midbrain tectum housing the PAG, is implicated in the regulation of the Hypothalamic–Pituitary—Adrenal Axis (HPA), often dysregulated in MDD. GABAergic interneurons selectively attenuate the firing of other neurons in the cortex through cortical inhibition (CI), crucial for several physiological functions in the cortex [331]. This suggests a potential mechanism through which the PAG could influence brain function and behavior, and potentially contribute to the pathophysiology of MDD.

The ACC plays a role in pain and emotional processing, which could be relevant to the discussion of the PAG. The ACC is involved in the affective aspects of pain, and its activity is increased in chronic pain states. This increased ACC activity is associated with anxiety phenotypes and can influence behavior in several ways, including altering reward responsivity in cost–benefit scenarios. Given the PAG’s role in visceral regulation and its connections to the medial prefrontal network, changes in ACC activity could influence the PAG’s function and contribute to the emotional dysregulation observed in MDD [325].

### Appendix C.17. Cerebellum (CB)

The cerebellum, despite its uniform cortical architecture, plays a role in numerous neurological functions—ranging from sensorimotor to emotional–social–psychological—with damage to different areas impacting these varied domains. Its interaction with extracerebellar structures provides heterogeneity to its function. The predictive coupling between grip and load force profiles has been interpreted within the theoretical concept of internal models for improved control. The cerebellum’s role in motor action predictions is crucial, given the lag associated with the cerebral motor cortex relying on gradually evolving sensory and somatosensory feedback, which could result in substantial time delays (∼100 ms). Thanks to its consistent anatomical structure—featuring one output cell and four primary interneurons—the cerebellum is well equipped to incorporate internal forward models. By employing reference copies of motor commands, the cerebellum, akin to a forward model, can anticipate the sensory impacts of voluntary actions, minimizing dependence on delayed somatosensory feedback.

The cerebellum’s architecture is often characterized as feed-forward in nature. This refers to the fact that information in the cerebellum flows in one direction, from input to output, without recurrent loops, in contrast to the cortex which has many recurrent connections [153]. This architecture supports its role in quick, real-time computations and predictions necessary for tasks such as motor coordination and possibly certain cognitive functions.

The cerebellum’s circuitry plays a key role in learning how to formulate accurate predictions by using error information about the differences between the actual and forecasted sensory consequences of voluntary actions. With its anatomical connections, the cerebellum is ideally positioned to compute expected sensory outcomes of voluntary motor actions for improved control.

Research from functional brain imaging further corroborates the cerebellum’s significance in establishing the equivalent of internal forward models within the central nervous system [332]. This concept can be generalized beyond the motor function. Through a computational process known as the universal cerebellar transform (UCT), the cerebellum integrates internal and external stimuli to maintain behavioral homeostasis. Dysmetria, a common symptom of cerebellum dysfunction, can manifest across all functional domains depending on the site of the lesion. This can result in motor, vestibular, or cognitive limbic symptoms—leading to different cerebellar syndromes [145]. Key computational principles of cerebellar function, like prediction- and error-based learning, are thought to operate cognitive cerebro-cerebellar loops [333]. Unlike the cerebral cortex, which depends on feedback connections, the cerebellum encodes internal models that predict and control a range of functions, from movement to cognition [104,145].

While prediction is a common function in many brain areas, cerebellar predictive capabilities are temporally constrained and temporally precise, representing *when*, as well as *what* is anticipated. The high temporal precision of cerebellar processing may be a key characteristic to differentiate cerebellar processing from that in the cerebral cortex [333]. The cerebellum, with its high level of convergence and reciprocal connections to much of the cerebral cortex, seems well positioned to integrate multiple inputs in order to generate estimates of future states, an idea formalized by the concept of the (forward) model. The cerebellum receives input from cortical areas via the pons and projects back to similar areas via the TH, forming a closed-loop architecture [333]. Resting-state functional connectivity studies highlight the interaction of the CB and HC, cingulate cortex, PFC, precuneus, and superior temporal cortex [333].

**Table A1:** Major systems in the brain and the regions or areas that fall into each.

System	Description	Regions/Areas
Cerebral Cortex	The outermost layer of the brain, involved in many complex functions including sensory perception, motor control, and higher cognitive functions.	Prefrontal Cortex, Insula, Primary Motor Cortex, Primary Sensory Cortex, Visual Cortex, Auditory Cortex, Parietal Cortex, Temporal Cortex
Limbic System	A group of structures involved in emotion, behavior, motivation, long-term memory, and olfaction.	Amygdala, Hippocampus, Thalamus, Hypothalamus, Cingulate Gyrus, Parahippocampal Gyrus
Basal Ganglia	A group of subcortical nuclei involved in control of voluntary motor movements, procedural learning, habit formation, and reward cognition.	Nucleus Accumbens, Caudate, Putamen, Globus Pallidus, Subthalamic Nucleus, Substantia Nigra
Thalamus	A large mass of gray matter responsible for relaying sensory and motor signals to the cerebral cortex and regulating consciousness, sleep, and alertness.	Anterior Nucleus, Medial Nucleus, Lateral Nucleus, Ventral Nucleus, Posterior Nucleus
Hypothalamus	A small region of the brain that plays a crucial role in many essential functions, including releasing hormones and regulating body temperature, circadian rhythms, and appetite.	Anterior Hypothalamic Area, Posterior Hypothalamic Area, Lateral Hypothalamic Area, Medial Hypothalamic Area, Mammillary Bodies
Midbrain	The middle division of the three primary divisions of the brain, involved in vision, hearing, motor control, sleep and wake cycles, arousal (alertness), and temperature regulation.	Ventral Tegmental Area, Periaqueductal Gray, Red Nucleus, Substantia Nigra
Cerebellum	A region of the brain that plays an important role in motor control, and may also be involved in some cognitive functions such as attention and language, and in regulating fear and pleasure responses.	Anterior Lobe, Posterior Lobe, Flocculonodular Lobe
Brainstem	The posterior part of the brain, responsible for basic vital life functions such as breathing, heartbeat, and blood pressure.	Midbrain, Pons, Medulla Oblongata

**Table A2:** Brain regions, their functions, associations with MDD, system association, functional network association, and corresponding algorithmic agent components. MDD: Major Depressive Disorder; DMN: Default Mode Network; NAN: Negative Affect Network; PAN: Positive Affect Network; SN: Salience Network; CEN: Central Executive Network.

Region	System	Description	Role in MDD	Agent Element	Functional Network
Prefrontal cortex (PFC), parietal, temporal, visual, sensory cortex	Cerebral Cortex	Involved in high-order cognitive processes such as decision-making, executive function, and emotional regulation.	Shows decreased activity in MDD, potentially contributing to problems with emotion regulation, decision-making, and cognitive control.	Modeling Engine	CEN
Insula/Insular cortex (IC)	Cerebral Cortex	Involved in consciousness, emotion, and the perception of bodily states (interoception).	Altered function in MDD may contribute to negative bias in emotion processing, as well as changes in the perception of internal bodily states.	Modeling Engine	SN
Hippocampus (HC)	Limbic System	Involved in learning and memory.	Shows reduced volume in MDD, potentially associated with memory problems and cognitive impairment.	Modeling Engine	DMN
Anterior cingulate cortex (ACC)	Limbic System	Part of the limbic system involved in emotion processing, learning, and memory.	Changes in function and structure in MDD may contribute to mood dysregulation, anhedonia, and cognitive problems.	Objective Function	DMN, SN
Amygdala (AMY)	Limbic System	Plays a key role in processing emotions, especially fear and sadness.	Shows increased reactivity to negative emotional stimuli in MDD, potentially contributing to the negative bias in emotion processing.	Objective Function	NAN
Nucleus accumbens (NAc), in ventral striatum (VS), basal ganglia	Reward System	Involved in processing reward and motivation.	Changes in function in MDD may contribute to anhedonia (loss of pleasure).	Objective Function	PAN
Paraventricular thalamus (PVT)	Thalamus	Involved in stress response and reward.	Alterations in function may be implicated in stress dysregulation and altered reward processing in MDD.	Objective Function (hub)	SN
Dorsolateral prefrontal cortex (dlPFC), ventromedial PFC (vmPFC), orbitofrotal cortex (OFC), anterior cingulate cortex (ACC), sgACC		Involved in executive function and emotional regulation.	Shows altered activity in MDD.	Planning Engine	CEN, SN
Hypothalamus (HYP)	HPA Axis	Plays a central role in maintaining the body’s internal balance or homeostasis, regulating hunger, thirst, body temperature, and sleep–wake cycles. Involved in reward processing and the responses to stressful and painful stimuli.	MDD is often associated with disruptions in the circadian rhythm, sleep, and appetite, potentially reflecting altered function.	Objective Function and Planning/action	SN
Ventral Tegmental Area (VTA)	Midbrain (part of Reward System)	Involved in reward, motivation, and the production of dopamine.	Changes in function could contribute to anhedonia and reduced motivation in MDD.	Planning Engine	SN

**Table A3:** Brain regions and corresponding interventions affecting them. Glossary: SSRIs (selective serotonin reuptake inhibitors), SNRIs (serotonin–norepinephrine reuptake inhibitors), TCAs (tricyclic antidepressants), MOIs (monoamine oxidase inhibitors), DBS (deep brain stimulation), TMS (transcranial magnetic stimulation), ECT (electroconvulsive therapy), tDCS (transcranial direct current stimulation), CBT (cognitive behavioral therapy), IPT (interpersonal therapy), CAM (complementary and alternative medicine), psychodynamic therapy (PSYDT).

Brain Region	Pharmacological Agents	Brain Stimulation	Psychotherapy	Lifestyle
Prefrontal Cortex (PFC)	SSRIs, SNRIs, TCAs, psychedelics	TMS, tES	CBT, IPT, PSYDT	Exercise, Mindfulness
Anterior Cingulate Cortex (ACC)	SSRIs, SNRIs, TCAs	TMS, tES	CBT, IPT, PSYDT	Exercise, Mindfulness
Amygdala (AMY)	SSRIs, SNRIs, Psychedelics	ECT	CBT, Psychodynamic Therapy	Mindfulness, Social Support
Hippocampus (HC)	SSRIs, SNRIs, TCAs	ECT, tES	CBT, IPT, Psychodynamic Therapy	Exercise, Sleep, Nutrition
Nucleus Accumbens (NAc)	SSRIs, SNRIs, MOIs	DBS, TMS	CBT, IPT	Exercise, Sleep, Nutrition
Ventral Striatum (VS)	SSRIs, SNRIs, MOIs	DBS, TMS	CBT, IPT	Exercise, Sleep, Nutrition
Ventral Tegmental Area (VTA)	SSRIs, SNRIs, MOIs	DBS	CBT, IPT	Exercise, Sleep, Nutrition
Paraventricular Thalamus (PVT)	SSRIs, SNRIs, TCAs, psychedelics	TMS, tDCS	CBT, IPT, PSYDT	Sleep, Nutrition
Hypothalamus (HYP)	SSRIs, SNRIs, TCAs	DBS, ECT	CBT, IPT	Sleep, Nutrition, Social Support
Insular Cortex (IC)	SSRIs, SNRIs, Psychedelics	TMS, tES	CBT, IPT, PSYDT	Exercise, Mindfulness
Brainstem	SSRIs, SNRIs, MOIs	DBS, ECT	CBT, IPT	Sleep, Nutrition, Social Support

## Appendix D. Relationship with the Free Energy Principle and AIF

KT is part of a family of information-centric theories of cognition and consciousness. This includes Predictive Coding or Processing Theory, Active Inference (AIF), and the Free Energy Principle (FEP) [39,113,206,334,335,336], alongside the anarchic brain and the entropic brain principle (REBUS/EBH) [112,255] and Integrated Information Theory (IIT) [337]. While these theories primarily draw from the Shannon information theory (statistics) and KT is rooted in algorithmic information theory, the divide between them can be bridged. Specifically, the pursuit of statistical regularities in data for probabilistic model construction can be interpreted as an effort to find the most concise program to describe it [338]. In this context, probability theory can be viewed as a subset of KT—potentially the most feasible approach for natural agents. As we will elucidate, KT offers a versatile mathematical framework with diverse implementation options, including avenues like AIF or IIT [339].

AIF is a formulation based on causal statistical theory that accounts for action, perception, and learning. The Free Energy Principle can be seen as a specific implementation of algorithmic principles integrating the Objective Function in the inference process using a Bayesian formulation. It states that in order to resist the natural tendency to disorder (i.e., maintain *stasis*), any adaptive change in an agent will seek to minimize free energy (surprise) or, equivalently, maximize the evidence for the generative internal model of sensory data. To support adaptation, the Bayesian agent must discover information about the likely external causes of sensory signals using its priors and information in the flux of the sensory signals coupled with actions. A key function required in the FEP formulation is the evaluation of prediction errors, which are then reused as data in hierarchical computations. Figure A1 provides a comparison of the KT and AIF agent models, highlighting the fact that in AIF, the evaluation of valence is implicitly carried out by the Comparator. Figure A2 highlights the hierarchical, distributed character of agent operations in natural systems.

To delve deeper into the connection between the FEP framework and KT, we must first acknowledge the key role of “surprise” within the FEP context. This metric gauges the extent of the agent’s prediction misalignment with the real-world data. Predictions stem from models informed by historical data and potentially inherent biases or priors. In mathematical terms, surprise is articulated as the negative log-likelihood of the data relative to the agent’s representation of the world, denoted as J=−logP(y). To diminish this element of surprise, the agent has the option to refine its models (like tweaking model parameters or a more fundamental model overhaul) or initiate actions that influence future data, thereby altering subsequent surprise levels.

Furthermore, the conceptualization of surprise within FEP inherently carries a bias. This manifests through the integration of homeostatic assumptions or priors into the model. For most natural agents, these are typically ingrained elements. They serve as a probabilistic representation of anticipated states of both the agent and its environment, leaning towards maintaining homeostasis. Consequently, the Objective Function inherent in KT is closely linked with FEP’s surprise concept.

In summary, AIF presents a unique architectural proposal for the instantiation of the abstract algorithmic agent model. In this framework, the Objective Function is evaluated as free energy within a Comparator mechanism. This represents a specific formulation of valence, integrating both the agent’s understanding of the world and its goals within the world. In contrast to KT, where prediction error is kept distinct from the Objective Function and may be used in combination with other factors, the FEP inherently combines these elements. The definition of FEP incorporates both “epistemic” error, which pertains to the agent’s understanding of the world (“what is the world like”?), and “pragmatic” error, which relates to the agent’s goals or desires (“what should the world be”?). This amalgamation of epistemic and pragmatic error is hardwired in the FEP, providing a specific design instance of the AIT agent model.

Finally, with regard to understanding MDD within the framework of AIF, several studies focus on disruptions in interoceptive predictions (modeling errors) as a source of a common vulnerability for mental and physical illness [157,208], while others take an evolutionary perspective to argue that depression is an adaptive response to perceived threats of aversive social outcomes to minimize prediction errors in interpersonal exchanges [340].

## Appendix E. Metaphysics, Experience and Mathematics

### Appendix E.1. Experience

In our efforts to understand the nature of consciousness, we often find ourselves grappling with a fundamental puzzle. Consider two distinct sets of atoms: one set constitutes a living, sentient entity, while the other forms an inanimate object. Prima facie, there appears to be an evident distinction between the two, primarily because one is capable of experiencing reality, while the other is not. One might argue that it is the unique arrangement of atoms in the conscious being that results in consciousness. But the counter-argument to this is the realization that, on a granular level, these atoms are identical to those in the inanimate set. So, why should one group be privy to the gift of consciousness and the other not? Furthermore, is the transition from consciousness abrupt or smooth as the arrangement of atoms changes? Either one creates new questions. When does it occur and why? If the change is smooth, what does it depend on and does it fall to zero at some point? Why?

What we are describing of course is the “hard problem of consciousness”, the “explanatory gap” [341], and the case for panpsychism, which is well explained in the work of Strawson and Goff, among others [342,343]. One way to motivate it is to note that experience exists, but this cannot be verified objectively. Science deals with measurement, i.e., a third-person view, while experience is a first-person phenomenon. Physics, as part of the scientific tradition, can never provide a mechanism for the abrupt appearance of consciousness. If a bunch of atoms do not have “experience”, it is hard to see how physics can explain why after some rearrangement it suddenly does.

It is much simpler to assume that experience is everywhere to begin with and address the problem of different “shapes” of experience, which may perhaps be related to the arrangement of atoms and their dynamics in a smooth way.

Experience must be brought into the picture as a first level class, an ontology at least as fundamental as “quantum field” or particle if physics is to be able to handle it in any way. This of course means merging first-person view with third-person, and redefining the meaning of science.

### Appendix E.2. Experience Everywhere

The notion that consciousness might exist only within the confines of (some?) biological entities is a deeply ingrained anthropocentric bias. Could we not posit that all matter possesses a degree of consciousness? This concept, known as *panpsychism*, presents a far less contradictory perspective. It proposes that all forms of matter, from the smallest subatomic particle to the vast galaxies, possess a rudimentary form of consciousness. This view does not attribute consciousness merely to the specific arrangement of atoms but instead suggests that consciousness is a fundamental and ubiquitous property of the universe.

The inherent beauty of panpsychism lies in its simplicity. It respects Occam’s razor, requiring fewer assumptions and circumventing the need for convoluted explanations. This philosophy does not dismiss the notion that higher levels of consciousness can emerge from more complex forms of matter. Instead, it acknowledges that every atom contributes its fragment of consciousness to the grand tapestry of existence.

Complementing panpsychism, we can also bring the concept of *idealism* into this discussion. Idealism posits that reality, as we know it, is fundamentally mental- or consciousness-based. It is a philosophy that places the mind, or, more broadly, consciousness, as the primary component of the universe. See Goff’s work for a lucid discussion on these philosophical notions [343].

In more detail, panpsychism and idealism are two distinct philosophical perspectives that approach the nature of reality and consciousness differently. Panpsychism posits that consciousness or a form of it, known as “experience”, is a fundamental and ubiquitous feature of reality. Panpsychists propose that every particle in the universe, from atoms to molecules, possesses a basic level of consciousness or experience. This does not necessarily mean that an atom has complex experiences like humans, but rather that it possesses some primitive form of experiential quality. The central idea is that consciousness is not exclusive to complex organisms like humans but is a universal property intrinsic to all matter.

Idealism, on the other hand, holds that reality as we know it is fundamentally mental- or mind-dependent. It asserts that all things that exist are constituted by mind or consciousness. In its most radical form, known as “subjective idealism”, it suggests that only minds and mental contents exist. A milder form, known as “objective idealism”, posits a shared reality that is made up of mental entities or is fundamentally dependent on them. The central idea is that consciousness or mind is not a product of physical reality, but instead, physical reality is a manifestation of consciousness or mind.

While both panpsychism and idealism attribute a central role to consciousness, they do so in different ways. Panpsychism considers consciousness as a fundamental property that all particles possess to varying degrees, while idealism regards the mental or conscious as the foundational substance of reality itself.

### Appendix E.3. Fields and Excitations

We can find parallels to the notion of structured experience in the domain of modern physics, particularly in quantum field theory (QFT). QFT describes the universe as a set of underlying quantum fields. Each fundamental particle, like an electron, is seen as an excitation within its respective field. All electrons are excitations in the same quantum field. The entire physical universe, in this model, arises from the “structured” excitations of a finite set of these quantum fields. The same applies to more complex structures, such as ferrets or humans.

Drawing a parallel with consciousness, we can consider the idealist idea of basic consciousness akin to these quantum fields. It presents the possibility that a form of consciousness permeates the universe, much like quantum fields do. Complex excitations or configurations within this universal consciousness field could then lead to what we perceive as individual structured experiences.

In this framework, the structured conscious experiences we have, as espoused by idealism, can be likened to the various manifestations of quantum fields. Just as the excitations and interactions within quantum fields give rise to the myriad forms in our physical universe, the structured computations within the consciousness field might give rise to the diverse array of subjective experiences we are privy to.

This interpretation acknowledges the indivisibility and oneness of all things at the most basic level while also recognizing the complexity and variety that emerge from this foundation.

#### Appendix E.3.1. Primordial vs. Structured Experience

The terrain of consciousness extends from the most fundamental, raw experiences to the intricate, organized perceptions we navigate daily. To appreciate this spectrum, we need to discern between two forms of experience: the primordial experience and the structured experience.

*Primordial experience*, as the term suggests, refers to the most basic, unadulterated state of consciousness. It is pure and undifferentiated, devoid of concepts, spatial–temporal organization, or a sense of self. This notion of unstructured consciousness is deeply embedded in Eastern philosophical traditions. In Buddhism, it corresponds to the state of “Shunyata” or emptiness, which lies beyond the realm of concepts and dualities. In Hindu philosophy, it resonates with “Brahman”, the ultimate reality that is beyond all classifications and qualifications.

*Structured experience*, on the other hand, represents the spatial, temporal, and conceptual organization of our first-person experience of the world and ourselves as agents within it [344]. This is where mathematics, “the science of structure, order, and relation that has evolved from counting, measuring, and describing the shapes of objects” [345], comes into play. It provides a framework to organize the raw, primordial experience into comprehensible structures, thus forming our everyday conscious reality. We refer to this phenomenon of consciousness endowed with such structure as “structured experience” [346]. It is the phenomenal structure of consciousness, the architectural design that shapes our perception and interaction with the world.

In our wakeful state, experience is typically structured to varying degrees. This structure emerges from the interplay of the primordial experience field with the mathematical principles governing spatial, temporal, and conceptual relations. From this perspective, we see consciousness not as a static, homogenous entity but as a dynamic spectrum extending from a pure, primordial experience to an intricate, structured experience. The dance between these two forms, choreographed by mathematics, breathes life into our diverse conscious experiences, transforming the raw potential of the experience field into the structured reality we perceive.

In KT, we start from the assumption that “there is experience” and only address the structural and valence features of experience associated with algorithmic features of agents. For this reason, there is no need for a magic threshold for the “emergence of consciousness”, unlike in other theories of consciousness.

#### Appendix E.3.2. Divided Experience

How is it that we only have access to our localized, divided experiences when there is a unique experience “field” encompassing all? This problem is strongly related to the “other minds” problem. One answer may stem from the notion that systems capable of structured experience and its reporting (to self or others) are localized agents equipped with basic interfaces, a Modeling Engine, and possibly an Objective Function for reporting valence. By definition, these systems are the sole entities that can access reportable, structured experiences, where experience transforms into algorithmic information. Consequently, sharing such experiences requires the creation, storage, and communication of this information, or *reports of experience*.

Structured experience seems inherently divided, but this perspective may be overly simplistic. Agents can form meta-agents, as cells in our body do, each presumably possessing its own structured experience. Why, then, do we lack awareness of these cellular experiences, and why do they not become a unified “I”? The answer lies in our differing coarse-grained models, which may not be communicable even if attempted. To elucidate this point, let us consider the following aspects.

- *Modeling:* A report of experience mandates a structure, thereby necessitating a model. Without a model, structure and meaningful reporting are impossible, explaining the lack of memories during infancy, where memory, a form of self-report, requires structured information.
- *Memory:* Even if a report is available, it may not be stored, resulting in amnesia. This absence of storage may occur for various neurobiological reasons, such as trauma, disease, or malformation of memory-related structures in the brain.
- *Communication:* If a model exists and is stored, its communication requires a common language. Without this shared language, interfaces, semantics, or proper channels, the model remains isolated.

The lack of such reports indicates either an incapability of generating them (modeling), storing them (memory), or communicating them (proper interfaces, common languages, and semantics). This may shed light on our relationships with other species, our brain subsystems, or inanimate matter.

However, the division of structured experience does not necessarily negate a union in primordial experience. Practices like meditation or religious traditions often aim to tap into this underlying unity. Furthermore, by sharing structured experiences through a common language, agents can partially transcend the division, sharing models, broadening and unifying our individual experiences.

### Appendix E.4. Mathematics and Experience

As we strive to grasp the essence of reality and consciousness, we encounter the intermingling domains of philosophy and physics. However, an intriguing facet that may enrich this conversation is the profound language of mathematics. Through its precise, abstract, yet widely applicable constructs, mathematics breathes life into our conceptual understanding of the universe. To visualize this, consider the idea of an all-encompassing “experience field” proposed in panpsychism, representing rudimentary consciousness pervading the universe. This field, analogous to the quantum fields in physics, underpins the myriad forms of conscious experiences we encounter.

However, this universal field of experience, much like the quantum fields, requires a sophisticated structure to manifest the intricate array of entities in our universe, from the simplest virus to the complex human intellect. This is where mathematics, with its inherent power to model and structure, steps in. Mathematics serves as the transformative catalyst, providing the structure and rules needed to sculpt the “experience field” into structured experiences. It is the mathematics that dictates how the excitations in the consciousness field interact, combine, and manifest into varying degrees of complexity, thus crafting the rich tapestry of consciousness we witness.

In a whimsical sense, we can imagine nature as the dream of a “mathematical dreamer”. The “experience field” is the dreamer’s psyche, and mathematics serves as the dreams themselves, shaping the raw material of the psyche into the tangible forms and experiences we recognize as reality. Through this lens, everything in existence becomes a harmonious dance between the “experience field” and mathematics. Consciousness or experience is not an isolated, fragmented phenomenon but a spectrum emerging from the mathematical orchestration of the universal experience field. It presents a perspective that unifies panpsychism, idealism, and quantum field theory, infusing them with the elegance and structure of mathematics. This view offers an intriguing framework to comprehend consciousness and its enigmatic relationship with the fabric of reality.

### Appendix E.5. Implications of KT for Non-Human Agents

The Kolmogorov theory (KT) of consciousness presents a broad-reaching perspective, where the scope of experience is universal, transcending the boundaries of human cognition and extending to non-human animal and artificial agents. According to the KT framework, any agent that constructs compressive models of the world possesses the capacity for structured experience. When an agent incorporates an Objective Function and demonstrates goal-oriented behavior, it can be said to possess a form of emotionality. This complexity endows the agent with a nuanced range of experiences that echo human emotions and mood states, making it susceptible to conditions akin to human depression.

The theory of natural selection further underscores the universality of the KT framework. The processes of evolution have endowed all evolved, living organisms with the capacity for a structured experience with valence. This experience stems from constructing a world model and evaluating that model against an Objective Function for planning. Natural selection necessitates an Objective Function in the organisms it shapes, driving them towards goal-oriented behaviors essential for survival and reproduction (stasis). Such a perspective suggests that emotions, and by extension, conditions such as depression, are not exclusive to humans but may be inherent in all forms of life shaped by natural selection. The nuanced interplay of world models, valence evaluation, and goal-oriented behaviors may thus represent a universal facet of evolved life.

This view expands the horizons of our understanding, indicating that we can potentially study these phenomena across various organisms. Furthermore, it also provides a fresh angle to consider the design and ethical implications of artificial intelligence, especially as we strive to create more sophisticated autonomous systems. Just as humans or other animals, sufficiently complex artificial agents might also experience forms of emotional distress or depression if their Objective Functions or goal-oriented behaviors are thwarted or persistently unfulfilled.

In essence, the framework suggests a universality of certain cognitive and emotional experiences, cutting across the divides between biological and synthetic agents. This idea prompts us to rethink not only our understanding of these conditions in humans but also how we design, interpret, and engage with artificial systems. Furthermore, it can potentially reshape our ethical considerations regarding the treatment and rights of both non-human and artificial agents.

These insights from the KT framework contribute to the ongoing discussions about consciousness, emotion, and mental health in the wider biological and artificial intelligence communities. They provide a new lens to view the interaction of cognitive structures, emotional experiences, and the potential for disorders akin to depression across a broad spectrum of agents.

## Appendix F. Embodied Cognition

In the context of MDD, we have taken the stance that the human agent is identified with the central nervous system (CNS). This means that the world outside the agent includes the agent’s body.

Alternatively, both brain and body can be taken to be part of the agent, in which case the body is seen as part of the agent system, processing external world data in various ways for the Modeling Engine, Objective Function, planner, etc. This corresponds to the view of “embodied” cognition. Both views are useful and complementary.

The boundary between agent and the external world is fuzzy and probably impossible to delineate mathematically. From the point of view of stasis, both the brain and the body can be seen as part of the agent since both are persistent as mathematical forms. But so is the ecosystem that they inhabit, which could, in turn, be seen as an extension of the body. In reality, we lack at present the mathematical formalism to define precisely “persistent algorithmic patterns”. Although this boundary is a matter of degree, one can say that the brain has more mutual algorithmic information with all the next layers [27].

In fact, one can reasonably argue that compressing data into a model commences at the sensory level. Information from the environment is selected, filtered, and compacted (i.e., lossy compression), preparing it for subsequent stages of modeling within the agent. Discerning which datum to utilize and the filtration method, i.e., *sensing*, is an integral part of model construction. Notably, the construction of such Modeling Engines is performed not merely by the individual agent but rather by the collective “class” of agents across multiple generations. Low-level data processing systems are shaped by evolution. This meta-agent (the true underlying persisting pattern), as it were, orchestrates the optimization of stasis over time, underscoring the intricate interplay between individual and collective learning in data compression and model building.

From this point of view, intelligence is not seen as a property of the individual agent alone but rather as a characteristic that emerges from the interaction between the agent’s body and its environment across generations to achieve *algostasis* (algorithm perdurance) or *stasis* for short (see the Glossary in Box 1). It underscores the idea that cognition is not merely a product of the brain but a holistic process involving the entire agent and its continuous interaction with the world. This perspective highlights the importance of considering both the individual and collective aspects of learning and adaptation, i.e., evolution, in our understanding of intelligence and cognition. When pushed to its logical limits, the theory suggests that the agent achieving stasis is, in fact, the entire system since all system components are integral to persistence and persisting. This view is consistent with modern ideas in systems ecology, such as the Gaia Hypothesis proposed by James Lovelock (1972), which holds that living organisms on the Earth interact with their surrounding inorganic environment to form a synergetic and self-regulating system that created, and now maintains, the climate and biochemical conditions that make life on Earth possible [347].

In this paper, we primarily focus on phenomena occurring over shorter timescales, where individual agents, or “organisms”, experience birth and death. Consequently, our attention will be centered on the more plastic elements of information processing within the agent within the central nervous system (CNS) instead of the body. Nevertheless, it is important to maintain an awareness of the broader perspective, where the pursuit of agent homeostasis, or of kin telehomeostasis, acts as the driving force. This longer-term view serves as a reminder of the overarching goal of “staying the same” in the face of environmental changes, a principle that in KT defines *life*.
